# Stem Cell Mechanobiology and the Role of Biomaterials in Governing Mechanotransduction and Matrix Production for Tissue Regeneration

**DOI:** 10.3389/fbioe.2020.597661

**Published:** 2020-12-14

**Authors:** S. M. Naqvi, L. M. McNamara

**Affiliations:** Mechanobiology and Medical Device Research Group, Department of Biomedical Engineering, College of Engineering and Informatics, National University of Ireland Galway, Galway, Ireland

**Keywords:** biophysical stimuli, 2D substrate stiffness, 3D biomaterial stiffness, biomechanical stimuli, tissue engineering, regenerative medicine, computational modeling

## Abstract

Mechanobiology has underpinned many scientific advances in understanding how biophysical and biomechanical cues regulate cell behavior by identifying mechanosensitive proteins and specific signaling pathways within the cell that govern the production of proteins necessary for cell-based tissue regeneration. It is now evident that biophysical and biomechanical stimuli are as crucial for regulating stem cell behavior as biochemical stimuli. Despite this, the influence of the biophysical and biomechanical environment presented by biomaterials is less widely accounted for in stem cell-based tissue regeneration studies. This Review focuses on key studies in the field of stem cell mechanobiology, which have uncovered how matrix properties of biomaterial substrates and 3D scaffolds regulate stem cell migration, self-renewal, proliferation and differentiation, and activation of specific biological responses. First, we provide a primer of stem cell biology and mechanobiology in isolation. This is followed by a critical review of key experimental and computational studies, which have unveiled critical information regarding the importance of the biophysical and biomechanical cues for stem cell biology. This review aims to provide an informed understanding of the intrinsic role that physical and mechanical stimulation play in regulating stem cell behavior so that researchers may design strategies that recapitulate the critical cues and develop effective regenerative medicine approaches.

## Introduction

While growth factors and the composition and surface chemistry of biomaterials have been widely adopted to control cell attachment, viability, protein adsorption, and differentiation of stem cells, the fate of stem cells is also intricately and intrinsically regulated by biophysical cues, which regulate proliferation, differentiation, gene expression, protein synthesis, matrix production, but also apoptosis and necrosis of the cells (Ingber, 2003). The field of mechanobiology is rapidly developing as appreciation of the importance of biophysical and biomechanical factors is growing and being adopted in the design of tissue regeneration studies. The focus of this specific review is to provide an informed perspective of how biomaterials govern differentiation of stem cells, with a particular focus on the role of mechanobiological factors presented by biomaterial substrates and 3D scaffolds in directing migration, self-renewal, proliferation and differentiation of stem cells. The initial focus is to present an introductory section providing important background into stem cell biology and mechanobiology, specifically focusing on the biological mechanisms by which stem cells can sense and interact with their surrounding mechanical environment. Next, the state of the art with respect to the current understanding that physical and mechanical cues play in controlling stem cell behavior is discussed. In this respect key experimental and computational studies are considered, which have unveiled critical information regarding how the physical and mechanical properties of biomaterials govern stem cell behavior. This review concludes by presenting a perspective on important unanswered questions and points to fundamental research that is still required to understand the intrinsic role of physical and mechanical stimulation in regulating stem cell behavior, and to design strategies that recapitulate these critical cues to develop effective tissue engineering and regenerative medicine approaches.

## Stem Cells

Stem cells play a critical role in tissue development and growth and repair throughout life. They are unspecialized cells and as such they do not have any tissue specific structures that allow them to perform specialized functions. However, they possess unique cellular characteristics defined by their capacity to either (1) undergo numerous cycles of cell division without differentiating (self-renewal) or (2) differentiate into specialized cell types (potency) in response to biochemical, biophysical and biomechanical cues. Stem cells undergo cell division either by means of Obligatory Asymmetric Replication, where one father stem cell divides into another father stem cell and one daughter cell or Stochastic Differentiation, where one father stem cell divides into two father stem cells and another father stem cell divides into two daughter stem cells (Lander, 2009).

**FIGURE 1:**
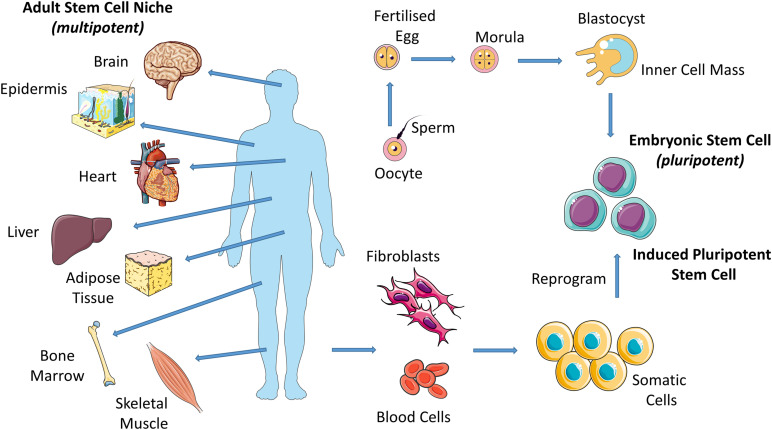
Eggs that have been fertilized *in vitro* give rise to embryos which give rise to embryonic stem cells (ESCs). Induced pluripotent stem cells (iPSCs) are adult cells that are genetically reprogrammed to a pluripotent stem cell–like state. Adult stem cells are undifferentiated cells, found among specialized cells in specific areas of adult tissues (called a “stem cell niche”). Pluripotent (ESCs and iPSCs) cells give rise to all cell types of the body and multipotent (adult stem cells) cells give rise to all cell types of a particular tissue or organ.

Stem cells are present in both embryonic and adult tissues but these exhibit different capacities for specialization. Stem cells that form the basis of the morula, the early-stage embryo that develops from the fertilized egg, are totipotent and have the capability to differentiate into all cell types including supportive extra-embryonic tissues such as the placenta. Totipotent cells give rise to pluripotent cells (i.e., embryonic stem cells (ESCs) from the blastocyst, late-stage embryo) which have the potential to differentiate into almost any cell type and thus have the potential to form all tissues in the body and ultimately construct a complete, viable organism (Figure 1; He et al., 2009). As the embryo grows, these stem cells continuously divide and become more specialized (Mitalipov and Wolf, 2009), losing their potency until they have the capacity to transform only into multiple cells from closely related tissues (multipotent cells). For example, hematopoietic stem cells (HSCs) can give rise to numerous types of blood cells, however, they cannot give rise to bone cells. Adult stem cells exhibit this limited potency. They are undifferentiated cells, found among specialized cells in specific areas of adult tissues (called a “stem cell niche”). There are several locations of adult stem cell niches, including brain, bone marrow, skeletal muscle, skin, heart, liver and fat (Figure 1). Adult stem cells can undergo self-renewal and can give rise to several specialized cell types surrounding their stem cell niche. In this way, adult stem cells maintain and repair their surrounding tissue. Adult stem cells are generally termed based on the tissue type they can regenerate; e.g., bone marrow stromal cells (BMSCs), HSCs and neural stem cells (NSCs). Adult stem cells may remain inactive (non-dividing) until they are required to maintain tissues or repair diseased or injured tissues, at which point they are activated.

Induced pluripotent stem cells (iPSCs) are used by many researchers in place of ESCs or adult stem cells due to limitations associated with their use. For example, ESCs, despite their pluripotency, display ethical concerns and adult stem cells exhibit a limited potency. Specialized adult cells are genetically reprogrammed to a pluripotent stem cell–like state (Figure 1; Singh et al., 2015). The defining properties of iPSCs, such as the potential to differentiate into almost any cell type, is maintained by way of forced expression of genes and proteins that are important for same.

Stem cells and progenitors are commonly cultured on (2D) or encapsulated within (3D) biomaterials for the purposes of large scale expansion, tissue regeneration or to enable fundamental studies of stem cell response to extracellular biochemical, biophysical and mechanical stimulation (Simmons et al., 2003; Luu et al., 2007; Mani et al., 2008; Wang et al., 2010; Fujita et al., 2014).

### Importance of Biophysical and Biomechanical Stimuli for Stem Cells *in vivo*

Biophysical stimuli are ever present within the human body and play a critical role in tissue formation from the earliest stages of embryogenesis and throughout life (Mammoto and Ingber, 2010). Physical and mechanical cues are important in embryonic tissue where ESCs self-renew and differentiate in response to these cues. In fact, it is established that mechanical forces are involved in patterning and organogenesis during embryonic development. The physical and mechanical environment of adult stem cells is also of great importance. Adult stem cells require cell-cell and cell-matrix interactions to maintain their potency.

While development of the embryo progresses, intrinsic forces exerted by cells transition from largely cell-cell in early-stage embryogenesis to more cell-matrix transmission as matrix content in tissues increases (Vining and Mooney, 2017). Concerted biochemical, biophysical and biomechanical cues work together to generate proper organ form. Mechanical forces in the early embryo (e.g., osmotic pressure, cell contractions, early muscle twitches) dictate cell viability, expression of genes important for development and organized cell movements, and atypical loading can lead to asymmetric development of embryonic rudiments (Beloussov and Grabovsky, 2006; Mammoto and Ingber, 2010). In limb development, early fetal muscle contractions are of great importance since biophysical stimuli precede local ossification and subsequent bone collar formation (Nowlan et al., 2008). Moreover, movements and muscular activity of the mother provide mechanical cues that are extremely important for normal development of the skeleton of a developing embryo (Carter, 1987; Carter et al., 1987). In the rudimentary heart of an embryo, cardiac cell contraction leads to tissue deformation and blood flow, which is critical to the normal development of a functioning heart with its chambers and valves (Goenezen et al., 2012).

The extracellular matrix (ECM) surrounding all cells in the body also exerts a mechanical influence that dictates cell phenotype, motility, biochemistry and matrix production. Throughout life maintenance of normal adult tissues relies on biophysical cues, and changes in the extracellular mechanical environment, or in the cellular mechanisms to sense such stimuli, have been associated with pathological conditions, such as hearing loss, muscular dystrophy, osteoporosis, osteoarthritis, cardiac myopathy, arteriosclerosis and age related degeneration (Jaalouk and Lammerding, 2009).

### Stem Cell Manipulation and Tissue Regeneration Strategies

Due to their capacity to self-renew and differentiate into specialized tissues, stem cells have been extensively studied to understand and take advantage of their ability to regenerate tissues for treatment of various human pathologies (Simmons et al., 2003; Luu et al., 2007; Mani et al., 2008; Wang et al., 2010; Fujita et al., 2014). Stem cells; isolated from embryonic and adult tissues, can be identified by the expression of various markers, such as CD13, CD29, CD44, CD54, CD73, CD90, CD105, CD146, CD166, and STRO-1 and by the absence of the markers CD10, CD11b, CD14, CD31, CD45, CD49d, and HLA-DR (Mafi et al., 2011). Stem cells can be either expanded or stimulated to differentiate into specific tissues (Schofield, 1978; Caplan, 1990, 1991; Jiang et al., 2002; Barry and Murphy, 2004; Caplan, 2005; Morrison and Spradling, 2008; Kuhn and Tuan, 2010; Morrison and Scadden, 2014) in the presence of biochemical cues, including growth factors, growth factor derivatives and peptide sequences, small bioactive molecules such as oxygen and nitric oxide and genetic regulators such as complimentary DNA, small interfering RNA and microRNA. These biochemical cues interact with stem cells through their receptors and, depending on the cue, they activate specific processes within the cell. The influence of growth factors and the composition and surface chemistry of biomaterials on stem cell biology has been extensively investigated, and it has been possible to control cell attachment, viability, protein adsorption and differentiation by these means. However, the fate of stem cells is also intricately and intrinsically regulated by biophysical cues, as is the activity of many biological cells, and these cues regulate proliferation, differentiation, gene expression, protein synthesis, matrix production, but also apoptosis and necrosis of the cells (Ingber, 2003). Biophysical environments include matrix architecture, topographical guidance, negative pressure, electrical stimulation, mechanical strength, electromagnetic therapy and surface morphology.

Various techniques have been developed to modify, control and assess the physical and mechanical properties of biomaterials for fundamental studies of stem cell biology and tissue engineering applications. Using such approaches, *in vitro* studies have sought to understand how biomaterial substrate (2D) stiffness regulates migration, proliferation and differentiation of stem cells. Tissue engineered scaffolds also provide distinct 3D physical and mechanical cues that regulate stem cell biology, but these differ from the bulk material behavior, due to the porosity, microarchitecture and nanoarchitecture of the scaffold. This is covered in further detail in section “Mechanobiological Responses of Stem Cells to Biophysical and Biomechanical Cues” of this review. Furthermore, computational models have been developed to provide a mechanistic understanding of the interaction between stem cells and the underlying biomaterial substrate or surrounding 3D scaffold. Such models can provide further insight into specific biological responses. This is covered in further detail in section “Computational Modeling of Cell-Biomaterial Interactions’ of this review. There is a distinct need to further understand mechanoregulatory cues that enhance stem cell differentiation to provide functional tissues for clinical applications.

## Mechanobiology: Mechanosensation and Mechanotransduction

**FIGURE 2:**
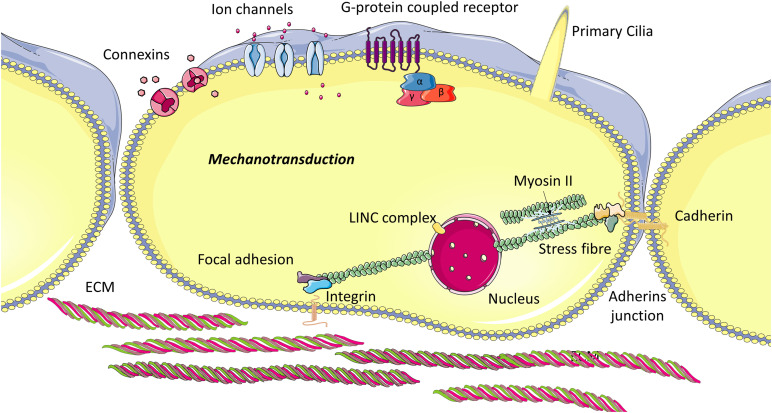
Cellular mechanosensory proteins: The internal cytoskeleton transmits mechanical stimuli from the extracellular environment to the cell nucleus. This stimulus is mediated by transmembrane proteins located at focal adhesions, which bind to ECM ligands but also intracellular proteins. Cadherins connect the cytoskeleton of adjacent cells and thus enable cells to transmit force from one to another, and also allow movement of components within the plasma membrane. Primary cilia sense fluid flow, pressure and strain and activate ion flux through channels on the ciliary axoneme, which govern intracellular signaling. Other membrane proteins can also be regulated through mechanical shear and strain.

Mechanobiology is an interdisciplinary field that integrates materials science and engineering mechanics with cell and molecular biology to investigate the mechanisms by which stem cells can sense (mechanosensation) and respond (mechanotransduction) to changes in their local mechanical environment. Stem cells are capable of monitoring their physical and mechanical environment by way of macromolecular complexes (Figure 2), known as mechanosensors, and initiate an adaptive response when the mechanical environment is not favorable. A comprehensive review published recently describes how stem cells sense mechanical stimuli in great depth and discusses how these cues are transduced into biochemical signals (Argentati et al., 2019). In this section, we briefly describe the mechanosensors identified to date and how stem cells respond to mechanical stimuli via these macromolecular complexes.

### Mechanosensation

A variety of macromolecular complexes have been identified and these include cytoskeletal related polymers and proteins (microtubules, f-actin microfilaments, intermediate filaments and actin-linking proteins), nucleoskeletal related proteins (SUN1, SUN2, lamins), adherens junctions (cadherins, α-catenin, β-catenin), focal adhesion proteins (vinculin), integrins, primary cilia, and ECM related proteins (fibronectin) (Bodle and Loboa, 2016; Argentati et al., 2019).

#### Cytoskeleton, Nucleoskeleton and Related Polymers and Proteins

The cytoskeleton links the nucleus to the ECM (Ingber, 1997) and thus can transmit mechanical stimuli from the extracellular environment. It is a dynamic structure that provides 3D support to cells and is responsible for, many if not, all cell functions (Harris et al., 2018). The geometry and polarity of its components (microfilaments, intermediate filaments and microtubules) influence cytoskeletal mechanical properties (Sun et al., 2010; Harris et al., 2018). Stem cells alter their internal cytoskeleton in response to external forces to reinforce and reorganize the cell, by actin polymerization or microtubule assembly, or alternatively to disassemble the cytoskeletal components and their transmembrane attachments to the ECM. Mechanical stimulation can activate the rearrangement of its components and lead to changes in cell morphology. This reorganization activates various intracellular signaling pathways (Wang et al., 2001), which are known to govern cell migration, proliferation and gene expression for tissue growth and function (McBeath et al., 2004; Engler et al., 2006; Wozniak and Chen, 2009). The interaction between the cytoskeleton and actin-linking protein myosin II (Sun et al., 2010) generates energy by means of ATP hydrolysis that move along the actin filaments and microtubules of the cytoskeleton to generate contractile forces on the ECM (Wang et al., 2001; Kollmannsberger et al., 2011; Crow et al., 2012). The regulation of cytoskeleton tension guarantees force propagation within cells (Wang et al., 2001; Kollmannsberger et al., 2011; Crow et al., 2012; Harris et al., 2018; Hamant et al., 2019).

Mechanical cues are transmitted through the cytoskeleton to the nucleus via the nucleoskeleton which is composed of the LINC (link the nucleoskeleton and cytoskeleton) complex (Hieda, 2019), Lamins A, B and C and other proteins such as lamina-associated polypeptides 2 (LAP2) and BAF. SUN domains and KASH domains make up the LINC complex (Luxton and Starr, 2014; Hao and Starr, 2019). The SUN domains associate with the nuclear lamina and the KASH domain bind to various cytoskeletal constituents. In this way, the LINC complex is a bridge that connects the lamina to the cytoskeleton (Wang et al., 2018; Hieda, 2019). Mechanical signals propagating through the LINC complex induce changes in gene and nuclear protein expression (Wang et al., 2018; Hieda, 2019). Another way mechanical cues are transmitted through the cytoskeleton to the nucleus is by structural modification of cytoplasmic proteins and their shuttling to the nucleus where they have a key role in regulating gene expression (Isermann and Lammerding, 2013; Cho et al., 2018). Among these proteins, YAP and TAZ (transcriptional coactivators) shuttle from the cytoplasm to the nucleus in response to increased stiffness (Aragona et al., 2013; Zanconato et al., 2016). In the nucleus, they bind transcription factors (e.g., RUNX, p73) and thereby control cell proliferation, apoptosis, and differentiation (Mo et al., 2014). Another protein, NKX-2.5 transcription factor shuttles from the cytoplasm to the nucleus in response to low tension. In the nucleus, it downregulates genes associated with maintaining a high-tension state (Cho et al., 2017).

#### Adherens Junctions and Gap Junctions

Cell-cell interactions allow mechanical forces to propagate across tissue cells. These interactions are coordinated by specific protein complexes such as Adherent Junctions (AJs). AJs play a significant role in remodeling of tissue, morphogenesis, wound healing, and tissue elongation (Takeichi, 2014; Khalil and de Rooij, 2019). The primary transmembrane cell-cell adhesion proteins forming AJs belong to the cadherin family. Nectins are another important membrane protein involved in the formation of AJs (Hirata et al., 2014; Takeichi, 2014; Griffin et al., 2017; Ishiyama et al., 2018).

Connexins are membrane proteins that can also be regulated through the mechanical environment (Thompson et al., 2012). Gap junctions are formed from two cellular hemi-channels composed of connexin proteins, which protrude through the cell membrane and connect the cytoplasm of adjacent biological cells. These channels are known to be sensitive to mechanical loading and permit movement of small molecules between adjacent cells and thereby initiate an intracellular signaling cascade in response to mechanical loading (Salameh and Dhein, 2013). Mechanical forces can varyingly modulate the expression and function of certain connections, such as Cx43. Gap junctional intercellular communication regulates proliferation, differentiation and apoptosis of stem cells (Wong et al., 2008).

#### Integrins and Associated Focal Adhesions

Bidirectional cell-matrix signaling between the inside and outside of the cells are mediated via the integrin family (De Arcangelis and Georges-Labouesse, 2000; Seetharaman and Etienne-Manneville, 2018; Mohammed et al., 2019). They are heterodimeric cell-surface transmembrane proteins that bind ECM proteins (ligands) to the cytoskeleton, but also facilitate interactions with other cells and act as signaling receptors (Hynes, 1992; Sawada et al., 2006; Arnaout et al., 2007; Ramsay et al., 2007). Integrins work in concert with the cytoskeleton to (1) perceive external mechanical stimuli, (2) facilitate movement by cells, (3) enable them generate tension on their extracellular environment, and (4) activate intracellular signaling pathways and elicit biochemical responses (Cary et al., 1999; Paszek and Weaver, 2004; Sawada et al., 2006; Arnaout et al., 2007; Puklin-Faucher and Sheetz, 2009).

Extracellular matrix proteins bind with integrins and activate intracellular proteins, such as those of the focal adhesion (FA) complex which transduces mechanical cues through this cell-matrix interaction and thus modulates cell functions (De Arcangelis and Georges-Labouesse, 2000). FA complexes are composed of a family of proteins, namely vinculin, paxillin, talin and focal adhesion kinase (FAK), which bind to the cytoplasmic domains of integrins via actin binding proteins (Ciobanasu et al., 2014; Martino et al., 2018). Depending on the mechanical cue the positioning and conformation of specific FA proteins can be altered (Kuo, 2013).

#### Primary Cilia

The primary cilium is a cellular organelle known to facilitate various physiological functions including development, photoreception, endocrine and exocrine function, renal function and chemical sensory processes (Davenport and Yoder, 2005). Primary cilia are important macromolecular complexes found in stem cells (Hoey et al., 2012b). Studies have discovered a primary cilium incidence of 60 – 85% in BMSCs (Tummala et al., 2010; Hoey et al., 2012b; Brown et al., 2014; Labour et al., 2016; Yuan et al., 2016; Corrigan et al., 2018; Johnson et al., 2018). Primary cilia are comprised of microtubule-based axoneme and encased in a plasma membrane that is continuous but distinct from the cell’s plasma membrane (Sorokin, 1962; Wheatley et al., 1996). Primary cilia extend into the pericellular fluid space and can also interact with the matrix. When the cilia is bent a Ca^2+^ influx occurs and spreads to neighboring cells (Schwartz et al., 1997; Praetorius and Spring, 2001; Praetorius et al., 2003). Of relevance here is the fact that the primary cilia play a role in sensing fluid shear stress in mesenchymal stem cells (MSCs). The presence of primary cilia affects biochemical responses to fluid flow applied to MSCs cultured in 2D (Hoey et al., 2012b) by upregulating osteogenic factors (Hoey et al., 2012a). It has been shown that the primary cilia mediates fluid flow mechanotransduction and ensuing osteogenic differentiation by hMSCs (Hoey et al., 2012b). In another study, the disruption of primary cilia in transplanted BMSCs (Kif3a knockout, a gene that is essential for primary cilia formation) demonstrated decreased bone formation in response to mechanical stimulation (Chen et al., 2016).

#### Extracellular Matrix (ECM)

The ECM is a structural macromolecular network that provides support for cells (Murphy-Ullrich and Sage, 2014; Theocharis et al., 2016; Stanton et al., 2019). It is composed of solid components (collagen, elastin, laminin, fibronectin, hyaluronic acid, chondroitin sulfate and syndecans) and soluble components (cytokines, growth factors, and matrix metalloproteinases and proteases), all of which serve as mediators between the cells and the ECM (Hynes, 2009; Murphy-Ullrich and Sage, 2014; Theocharis et al., 2016; Mohammed et al., 2019; Stanton et al., 2019). There are two main types of ECM that differ with regard to their structural organization and composition: the connective tissue provides a 3D scaffold and the basement membrane provides 2D support (Hynes, 2009; Frantz et al., 2010; Janson and Putnam, 2015; Mohammed et al., 2019). The ECM is composed of fibers, proteoglycans (PGs), and glycoproteins. Topography, viscosity, and mechanical properties of the ECM are determined primarily by the amount, type, and arrangement of these macromolecules. As such, the ECM may have characteristics of a soft material or a stiff material (Jansen et al., 2015). Stem cells may secrete ECM structural components and matrix metalloproteinases, or exert mechanical forces through the cytoskeleton fibers and in these ways may change the ECM composition and remodel the architecture.

### Mechanotransduction

Using various techniques, mechanical forces have been applied to ESCs (Yamamoto et al., 2005) and adult stem cells harvested from bone marrow (Grellier et al., 2009), fat (Hanson et al., 2009), and tendon (Zhang and Wang, 2010) and results show that stem cells are sensitive to their mechanical environment. Stiffness can be modified by several means. Crosslinking during polymerisation is widely used to alter biomaterial physical and mechanical properties and refers to the degree of bonds between molecules which can be modulated through the use of biochemical crosslinkers, exposure to ultraviolet light, photopolymerization, enzymatic reactions or by altering pH, temperature or the ionic environment (Drury and Mooney, 2003; Chau et al., 2005; Tierney et al., 2009; Keogh et al., 2010; Haugh et al., 2011). ECM stiffness can also be altered by coating cytotoxic polymers with cell adhesive ligands such as collagen (Evans et al., 2009), laminin (Rowlands et al., 2008), and fibronectin (Rowlands et al., 2008; Altmann et al., 2011). Substrate stiffness experienced by the cell may be controlled by varying the substrate thickness (Sen et al., 2009; Leong et al., 2010). Micropost arrays (MAs) can also be used to present various mechanical rigidities for the purposes of studying mechanobiology (Tan et al., 2003). The role that substrate stiffness plays in stem cell biology is covered in further detail in section “Mechanobiological Responses of Stem Cells to Biophysical and Biomechanical Cues” of this review. The changes in gene and protein expression in mechanically stimulated cells involve the production of biochemical signals, which is known as mechanotransduction. The forces experienced by stem cells and their mechanosensory macromolecular complexes activate specific signaling pathways, which transduce mechanical messages into actions within the cells, such as production of growth factors and synthesis of ECM proteins. There are various mechanotransduction signaling pathways, and these rely on the interaction of intracellular ions and molecules, which undergo concentration changes due to the mechanical stimulus. There are numerous downstream signaling events that are activated, namely YAP/TAZ signaling, Rho/ROCK signaling, FAK, mitogen activated protein kinase (MAPK) and G protein related and calcium signaling (Castillo and Jacobs, 2010).

#### YAP/TAZ Signaling

YAP/TAZ is a mechanosensitive intracellular signaling pathway that mediates stem cell biology through upstream signaling pathways including Hippo, Smad, Wnt, G-proteins, and MAPK, which is comprehensively reviewed in Shao et al. (2015). YAP and TAZ transduce signals important for driving stem cell fate. YAP/TAZ has been shown to localize to the nucleus in MSCs cultured on stiff substrates (40 kPa) or allowed to spread, and these cells underwent osteogenic differentiation, whereas YAP/TAZ remained in the cytoplasm for those cells cultured on a soft substrate (0.7 kPa) or an environment that induces cell rounding, and these were shown to undergo adipogenesis (Dupont et al., 2011). Their abnormal activity is involved in in several diseases such as atherosclerosis, fibrosis, pulmonary hypertension, inflammation, muscular dystrophy, and cancer (Panciera et al., 2017).

#### Rho/ROCK Signaling

Rho/ROCK signaling and cytoskeleton tension have been shown to be important for mechanotransduction in stem cells (Tenney and Discher, 2009). Activated Rho (Paszek et al., 2005) promotes actomyosin stress fiber assembly in response to increased stiffness (Chrzanowska-Wodnicka and Burridge, 1996), significantly changing the mechanical properties of the cell (Hall, 1998). Stem cells exhibit dissociation-induced apoptosis (Thomson et al., 1998; Zhang et al., 2011) which is caused by actomyosin hyperactivation through the Rho/ROCK pathway (Chen et al., 2010; Ohgushi et al., 2010). In the presence of a ROCK inhibitor; the survival and cloning efficiency is increased in stem cells (Watanabe et al., 2007; Zhang et al., 2011). In addition to actomyosin stress fiber assembly, activated Rho in response to increasing stiffness leads to increased cell contractility and/or the activation of pERK, which enhances osteogenic differentiation (Arnsdorf et al., 2009). Inhibition of Rho/ROCK signaling enhances adipogenic or chondrogenic differentiation. Rho/Rock signaling is also required for MSC tenogenic differentiation. Disruption of the cytoskeleton and the Rho/ROCK pathway of MSCs on rope-like silk scaffolds diminish the expression of tendon differentiation markers and lead to a loss of spindle morphology (Maharam et al., 2015). Furthermore, downregulation of osteogenic marker RUNX2, mediated via the Rho/ROCK signaling pathway promotes the differentiation of dental pulp stem cells into odontoblasts (Huang et al., 2018). Rho and the actin cytoskeleton have also been shown to be necessary to maintain nuclear YAP/TAZ in MSCs (Dupont et al., 2011).

#### Focal Adhesion Kinase (FAK)

Focal Adhesion Kinase regulates human adipose stem cell (hASCs) differentiation via ROCK signaling (Hyväri et al., 2018). Active FAK and ROCK resulted in upregulation of osteogenic marker *RUNX2A*, increased ALP activity and matrix mineralization implicating osteogenesis. Inhibition of FAK and ROCK activity resulted in upregulation of adipogenic markers *AP2* and *LEP* and lipid accumulation implicating adipogenesis. Another study demonstrated that compressive stimulation (2 g/cm^2^) upregulated COX-2 expression and increased phosphorylated FAK and prostaglandin E(2) (PGE2) in human periodontal ligament cells (hPDL). In this way, FAK regulates hPDL cells via COX-2 expression and the associated production of PGE2 under compression (Kang et al., 2010).

#### MAPK

MAPKs are a family of enzymes (ERK1/2 and ERK5, JNK1/2/3 and p38α, p38β, p38γ, and p38δ) that are implicated in a series of mechanotransduction pathways (Cargnello and Roux, 2011). ERK has been implicated as a regulator of differentiation in stem cells. In addition to biochemical stimuli, mechanical forces also activate ERK through integrin focal adhesion complexes and the MAPK-ERK signaling cascade (MacQueen et al., 2013). Mechanical stimulation increases matrix mineralization with MSCs in osteogenic differentiation media via the ERK pathway (Simmons et al., 2003). In addition to ERK, p38 is also implicated as a regulator of differentiation in stem cells. The p38-MAPK signaling cascade has demonstrated to be essential for skeletogenesis and osteoblast differentiation (Greenblatt et al., 2010; Thouverey and Caverzasio, 2012; Rodríguez-Carballo et al., 2014). Furthermore; mechanical loading (stretching, compressive force and fluid shear) has been shown to induce osteogenic differentiation via p38-MAPK activation (Kreke et al., 2008; Kim et al., 2010).

#### G-Protein Related and Calcium Signaling

G-protein-coupled receptors (GPCRs) at the cell membrane level are also involved in mechanotransduction (Sarasa-Renedo and Chiquet, 2005; Vogel and Sheetz, 2006; White and Frangos, 2007). One study, performed in rats, demonstrated that G-protein Neuropetide Y (NPY), through GPCR Y1 activation, is a proliferative regulator of rat NSCs (Thiriet et al., 2011). A comprehensive review discusses the role of GPCRs in the regulation of stem cells in more detail (Doze and Perez, 2013).

Another consequence of applying mechanical force to the cell surface is a change in calcium (Ca^2+^) influx through stretch-activated channels (Wu et al., 1999). This alteration in the Ca^2+^ influx may lead to the activation of MAPK signaling pathway (Rosen and Greenberg, 1996; Sadoshima and Izumo, 1997; Iqbal and Zaidi, 2005).

## Mechanobiological Responses of Stem Cells to Biophysical and Biomechanical Cues

Biomaterial matrices can maintain pluripotency and suppress differentiation, or can be used to encourage differentiation (Engler et al., 2006; Khatiwala et al., 2006). Biomaterials can be produced from natural polymers (collagen, hydroxyapatite, alginate, chitosan or cellulose derivatives) or synthetic polymers (polyvinyl alcohol, polyethylene glycols (PEG), poly(lactide-coglycolide)) (Vinatier et al., 2009). The choice of biomaterial is critical to the cellular behavior, and a vast array of studies have sought to identify specific features of the biomaterial, including composition, surface topography, ligand availability, and mechanical properties, which influence cell migration, proliferation, differentiation and viability (Table 1) (Engler et al., 2006; Huebsch et al., 2010; Abdeen et al., 2016). Mechanical stimulation also plays an important role in directing responses of stem cells *in vitro* (Rubin et al., 2007; Sen et al., 2008; Arnsdorf et al., 2009; Potier et al., 2010; Case et al., 2011; Gurkan et al., 2011).

**TABLE 1:** Key Studies of the effect of 2D substrate stiffness, substrate thickness, substrate rigidity gradients, and 3D biomaterial stiffness on stem cell behavior.

Experimental approach	Key findings	References
**2D biomaterial substrates**		
**Substrate stiffness**		
**Crosslinking during polymerisation**		
PA substrates and Collagen I gels with varying thickness (>10 μm – 500 μm)	For PA substrates, MSCs on stiff (15 kPa) exhibit higher expression of SMC markers and on soft (1 kPa) exhibit increased chondrogenic (collagen-II) and adipogenic (LPL) marker expression. For collagen I gels, hMSCs on thick gels (soft) had lower expression levels of SMC markers than on a thin (stiff) substrate and chondrogenic marker (collagen II) increased in hMSCs grown on thick gels (soft).	Park et al., 2011
PA hydrogels with controlled presentation of peptides	Stiff (10 kPa) substrate activated YAP/TAZ nuclear localisation in hESCs. Soft (0.7 kPa) substrate exhibited low levels and diffuse cytoplasmic staining of YAP/TAZ.	Musah et al., 2012
Fibronectin coated acrylamide hydrogel	Osteogenic differentiation of BMSCs on stiff (15–40 kPa) substrate was inhibited by (1) depletion of YAP and TAZ, (2) culturing cells on soft ECM (0.7–1 kPa) or (3) incubating with a Rho inhibitor (C3). YAP and TAZ knockdown allowed adipogenic differentiation on stiff substrates by mimicking a soft environment.	Dupont et al., 2011
Collagen coated HA hydrogels and PA hydrogels	Increased expression of mature cardiac markers and muscle fibers when pre-cardiac cells seeded on stiff hydrogels (collagen coated HA) (1.9 – 8.2 kPa) compared to compliant PA hydrogels.	Young and Engler, 2011
Collagen I coated PDMS substrate (0.041 – 2.7 MPa)	Osteogenic differentiation (OPN and RUNX2) and mineralisation by ESCs was enhanced on stiff substrates (>2.3 MPa) when compared to soft substrates (0.04 – 1.9 MPa). Genes expressed in early mesendoderm differentiation were also upregulated on stiff substrates. Cell spreading and growth increased as a function of substrate stiffness, whereas cell attachment was unaffected.	Evans et al., 2009
Tropoelastin substrates (stiffness not reported)	Mouse HSCs and hHSCs cultured on tropoelastin enhanced expansion and maintenance of undifferentiated cells. Substrates cross-linked with >0.1% glutaraldehyde to alter the elasticity, the biological effects of tropoelastin were lost. Mechanotransduction inhibition also abrogated these effects.	Holst et al., 2010
**Photopolymerisation**		
Photopolymerisable methacrylamide chitosan substrates	Neural stem/progenitor cells were most proliferative on soft substrates (3.5 kPa). Neuronal differentiation was favored on the softest surfaces (< 1 kPa). Oligodendrocyte differentiation occurred on stiffer surfaces (>7 kPa). Astrocyte differentiation was only observed in small percentage on < 1kPa and 3.5 kPa surfaces.	Leipzig and Shoichet, 2009
**Ligand availability**		
PA substrates with different ligand coatings	Osteogenic differentiation of MSCs (RUNX2) increased with substrate stiffness (0.7 kPa to 80 kPa), and occurred significantly only on high stiffness collagen I coated gels (80 kPa). Collagen IV, fibronectin or laminin I coated substrates stimulated osteogenic differentiation when the stiffness was ∼25 kPa. Myogenic differentiation occurred on all gel-protein combinations that had stiffness >9 kPa, but peaked for fibronectin coated gels with a modulus of 25 kPa.	Rowlands et al., 2008
Matrigel coated PA hydrogels	Increasing ECM stiffness increased hPSCs and colony spread area but did not alter self-renewal, in contrast to mESCs. Soft matrices (100 – 700 Pa) promoted expression of early neural ectoderm markers, and downstream increases in total neurons and dopaminergic neurons.	Keung et al., 2012
Ligand (fibronectin, collagen I, collagen IV and laminin) coated PA hydrogels	YAP nuclear translocation occurred at low density (5 μg/mL) for fibronectin, collagen I and collagen IV and at a higher density (20 μg/mL) for laminin, when cultured on stiff hydrogels. Low ligand densities (cytoplasmic YAP localization for all ECM types) results in low osteogenic commitment (RUNX2 and ALP), for cells on hydrogels coated with collagen I or fibronectin. High ligand densities, (nuclear YAP localization for all ECM types) results in high osteogenic commitment for cells on all ECM types except collagen IV.	Stanton et al., 2019
Laminin coated PEG hydrogel (2 – 42 kPa)	Muscle stem cells cultured on soft PEG hydrogels (12 kPa), with stiffness close to native muscle elasticity, promoted self-renewal *in vitro* and enhanced muscle regeneration when transplanted into mice. This was not observed on stiff tissue culture plastic (∼106 kPa). The migration velocity of the stem cells increased (120 μm/h) when they were cultured on the stiff PEG hydrogels when compared to softer matrices (99 μm/h).	Gilbert et al., 2010
PEG hydrogels with varying concentrations of RGD (0.05–2.5 mM)	Human MSCs seeded on soft hydrogels (7.4–11.2 kPa) clustered with reduced cell attachment and spreading area, irrespective of RGD concentration and isoform. Human MSCs seeded on stiff hydrogels (27.3–36.8 kPa) spread with high spatial coverage for RGD concentrations of ≥ 0.5 mM.	Chahal et al., 2018
Collagen coated PDMS (soft, 0.07–0.10 kPa, stiff, 2.15–2.40 MPa)	Diminished hMSC contractility on soft substrates of hydrophobic PDMS and hydrophilic polyethylene-oxide-PDMS (PEO-PDMS). Cell spreading and osteogenic differentiation occurred only on soft hydrophobic PDMS and not on soft hydrophilic PEO-PDMS (elastic modulus < 1 kPa)	Razafiarison et al., 2018
**Carbon nanotubes**		
Glass surface densely coated with carbon nanotubes	BMSCs exhibit high ALP activity, upregulation of osteogenic markers (BMP2, RUNX2, ALP and OCN) and increased calcium content.	Mori et al., 2020
**Substrate thickness**		
PA substrates (0.1 – 40 kPa)	MSCs on the softest substrates (0.1 – 1 kPa) demonstrated a branched morphology and expressed neurogenic markers. MSCs on the intermediate stiffness substrates (8 – 15 kPa) displayed a spindle like morphology and expressed myogenic markers. MSCs on substrates stiffest substrates (15 – 40 kPa) adopted a spread morphology and expressed osteogenic markers.	Engler et al., 2006
Collagen coated PA gels	MSCs on 0.5 mm thick gels exhibited the same spread morphology as those cultured on collagen substrates of 34 kPa. MSCs on thicker substrates (2 mm) of identical composition behaved similarly to MSCs cultured on 1 kPa collagen gels.	Amnon et al., 2010
Wedge shaped gels	The focal adhesion area decreases as substrate thickness increases (up to 5 μm thickness) and cell induced forces travel only a limited distance (micrometers) through linear, homogenous substrates such as PA.	Maloney et al., 2008
**Substrate rigidity gradients**		
Human ASCs on PA substrates with stiffness gradients (0.5, 1.7, 2.9, 4.5, 6.8, and 8.2 kPa/mm) at the cell–matrix interface	Stiffness gradients of 2.9 kPa/mm were found to be nondurotactic. Durotaxis was observed on matrices with gradients of 8.2 kPa/mm. Lamin A expression scaled in a dose-dependent manner in response to stiffness, and Lamin A/Lamin B ratios increased exponentially with stiffness. The MRTF-A was affected by stiffness and peaked at ∼20 kPa. Adipogenic marker PPARγ was upregulated at 3 kPa, myogenic transcription factor MyoD was upregulated at 12 kPa, whereas the osteogenic marker CBFA1 was highest at 36 kPa.	Hadden et al., 2017
Microelastically patterned gels	The threshold stiffness gradient (TG) (0.14 to 1.4 kPa/μm) for hMSCs markedly increased with an increase in the absolute stiffness (2.5 to 10 kPa) of the soft region, attributed to more stabilized focal adhesions in the stiffer soft region. The intrinsic stiffness gradient (IG) of the material should exceed position-dependent TG to induce durotaxis.	Moriyama and Kidoaki, 2019
**Substrate stiffness and porosity**		
PA substrate (4 – 33 kPa)	Varying porosity did not significantly change matrix tethering, substrate deformations or stem cell differentiation potential. Osteogenic and adipogenic differentiation were unaffected by varying the protein–substrate linker density or in the absence of protein tethering	Wen et al., 2014
PA hydrogel surfaces (0.5 740 kPa) and Collagen coated PDMS surfaces (0.1 kPa–2.3 MPa)	For the PA surfaces, pore size was inversely correlated with stiffness (15 nm in 2 kPa gels, but >2 nm for gels >115 Pa). Epidermal stem cells remained rounded and underwent terminal differentiation on high porosity substrates (soft), whereas cells spread and remained undifferentiated on low porosity substrates (stiff). For collagen coated PDMS surfaces of low nanoparticle density (190 nm spacing), keratinocytes differentiated and did not spread, but on collagen coated PDMS with closely anchored nanoparticles (60 nm) cells spread and did not differentiate.	Trappmann et al., 2012
**Switching Stiffness – mechanical memory**		
Fibronectin coated PA hydrogels	MSCs cultured on soft hydrogels (∼0.5 kPa) expressed markers for neurogenesis whereas those cultured on stiff hydrogels (∼40 kPa) expressed increased markers of osteogenesis. Transfer of MSCs to hydrogels of the opposite stiffness resulted in a switch in lineage specification. MSCs originally cultured on stiff hydrogels maintained increased markers of osteogenesis.	(Lee et al., 2014)
PA substrate	ASCs behaved similarly to BMSCs by committing to becoming neurogenic, myogenic, and osteogenic on 1, 10, and 34 kPa. ASCs fused into multi-nucleated myotubes, expressed mature muscle proteins and remained fused when switched to a stiff niche.	Choi et al., 2012
Photodegradable PEG hydrogels	Activation of YAP/TAZ and RUNX2 in hMSCs cultured on soft substrates (2 kPa) depended on previous culture time on stiff substrates (3 GPa). Human MSCs cultured initially for short durations (>7 days) on stiff hydrogels (∼10 kPa), followed by culture on soft hydrogels (∼2 kPa) demonstrated reversible activation of YAP/TAZ and RUNX2. This activation was irreversible in cells cultured for 10 days on stiff hydrogels before further culture on soft substrates. Increased durations of culture for MSCs on stiff tissue culture polystyrene enhanced osteogenic differentiation.	Yang et al., 2014
Methacrylated HA substrates	Human MSCs switched from adipogenic to osteogenic differentiation upon *in situ* substrate stiffening (soft (3 kPa) to stiff (30 kPa)). These changes were accompanied by increases in cell area, traction forces and motility, which equilibrated within 2–4 h. Early switching (minutes-to-hours) favoured osteogenic differentiation of the hMSCs and later switching (days-weeks) tended towards adipogenesis.	Guvendiren and Burdick, 2012
Polyelectrolyte multilayer coated shape memory polymer	Human iPSC-derived cardiomyocytes (CM) had no preferential directionality within 0 to 12 h, and slowly reoriented to the wrinkle direction starting at h 16. The cell aspect ratio slightly increased from h 16. The sarcomere index reduced at h 4-8, thin filament length increased within h 8-24, the sarcomere length increased within h 16-24 compared to h 0 and vinculin length decreased at h 4 and 8 but returned to the original length at h 12.	Sun et al., 2020
**Micropost arrays**		
PMAs	Osteogenic differentiation was favoured on rigid PMAs (*K* = 1,556 nN μm^–1^) whereas adipogenic differentiation was enhanced on softer PMAs (*K* = 1.90 nN μm^–1^). Human MSCs that underwent osteogenic differentiation demonstrated higher traction forces than non-differentiating cells. Human MSCs that did not differentiate into adipocytes were more contractile than differentiating adipocytes. Osteogenesis of hMSCs was decreased following Rho-ROCK inhibition.	Fu et al., 2010
Vitronectin coated PMAs (1.92 kPa – 1,218.4 kPa)	>20% of hESCs cultured on rigid PMAs remained undifferentiated compared to cells on soft PMAs. Human ESCs were shown to increase cytoskeletal contractility with increased matrix rigidity.	Sun et al., 2012
PMAs (5 kPa – 1,200 kPa)	Soft substrate (5 kPa) promoted hESC neuroepithelial conversion. Purity and yield of functional motor neurons derived from neural progenitors was enhanced on soft PMAs.	Sun et al., 2014
**3D biomaterial scaffolds**		
Cross-linked HA and PA substrates (0-40 kPa)	Stiffer matrices promote MSC spreading. MSCs embedded in HA matrices were constrained to spherically symmetric shapes and the assembly of cortical cytoskeleton. Inhibition of myosin-II contractility (using Blebbistatin) prevented spreading of MSCs.	Rehfeldt et al., 2012
Fibronectin-hyaluronic acid (FN-HA) 2D substrates and 3D hydrogels	Human MSCs experience an increase in nuclear translocation of YAP when cultured on 2D substrate with increasing amounts of FN hydrogel while the stiffness (7 kPa) remained constant. This is not observed for MSCs encapsulated in 3D hydrogels.	Trujillo et al., 2020
PEGDM polymers	Osteogenic differentiation occurred predominantly when MSCs were encapsulated within moderate stiffness 3D hydrogels (11–30 kPa), whereas adipogenesis was favored for hydrogels within the 2.5–5 kPa stiffness range.	Huebsch et al., 2010
Covalently crosslinked HA matrices (4.4 – 91 kPa)	MSCs undergo adipogenic differentiation when they are encapsulated within non-degradable matrices, whereas osteogenic differentiation was observed in HA matrices that were modified to be degradable. Within hydrogels of the same modulus, osteogenesis was favored when cells were able to contract the surrounding matrix, whereas adipogenesis was favored when cells were restricted to be rounded by secondary physical crosslinking.	Khetan et al., 2013
Collagen scaffolds (1, 2, 7 and 29.7 kPa)	Osteocalcin and perilipin were both found intracellularly. Osteocalcin signal intensity per hBMSC was greater in the medium-stiffness compared to the low and high stiffness scaffolds. Perilipin signal intensity decreased with increasing stiffness.	Herrera et al., 2019
Void forming nano-porous hydrogel	Cell proliferation and osteogenic differentiation (ALP) were shown to peak in void-forming hydrogels with intermediate bulk stiffness (60 kPa), but drop off for those at higher stiffness (110 kPa). Collagen I expression and mineralization by MSCs within void-forming hydrogels were also shown to be enhanced in hydrogels with a bulk elasticity > 60 kPa.	Huebsch et al., 2015
Macro-porous substrates	MSCs upregulated markers for both osteogenesis (ALP) and adipogenesis (triglyceride) when cultured in stiff 3D porous substrates (16, 50 kPa), when compared to soft counterparts (0.5 kPa)	Haugh et al., 2018
3D ECM-like fibrous structures	Smallest pore size (100 μm) was optimal with the greatest stiffness, greatest seeding efficiency, maintenance of spread cell morphology and significantly greater collagen and calcium deposition.	Brennan et al., 2019
Viscoelastic alginate hydrogel	Viscoelastic alginate hydrogels that exhibited fast stress relaxation were shown to enhance cell spreading, proliferation, osteogenic differentiation by MSCs and formation of a mineralized matrix.	Chaudhuri et al., 2016.
Viscoelastic alginate hydrogels	A reduced rated of stress relaxation or an increased osmotic pressure restricts volume expansion and reduces osteogenesis, regardless of cell morphology. A reduced osmotic pressure induces volume expansion and accelerates osteogenesis.	Lee et al., 2019
GelMA hydrogels	Elevated elasticity surrounding hASCs embedded in soft hydrogels. Greater elasticity (>10 kPa) in GelMA containing TAZ-activated-hASCs.	Hepburn et al., 2020
Microphotopatterning (μPP) substrates	Spontaneous Ca^2+^ oscillations in hMSCs during collagen matrix assembly. Inhibition of TRPV4 reduced Ca^2+^ signaling, reduced aligned collagen fibril assembly and decreased tensile force across vinculin. Activating TRPV4 accelerated aligned collagen formation and caused a dynamic unloading and reloading of vinculin. TRPV4-dependent Ca^2+^ oscillations were found to be independent of pattern shape or subpattern cell location.	Gilchrist et al., 2019

Biomaterial mechanical properties have been characterized by several means such as atomic force microscopy (AFM). AFM has been widely used to characterize the mechanical properties of soft biological substrates, tissues and cells (Engler et al., 2006; Evans et al., 2009; Huang et al., 2013; Mullen et al., 2013, 2014, 2015; Pietuch and Janshoff, 2013; Wen et al., 2014; Mc Garrigle et al., 2016).

### 2D Substrates

The biophysical properties of biomaterial substrates have been investigated using numerous methods to derive an understanding of specific properties that could control stem cell behavior. Many researchers have demonstrated that differentiation, morphology and motility in stem cell types is dictated by the stiffness of substrates onto which cells are seeded. For example, aorta-derived smooth muscle cells increased spreading with increased stiffness (Engler et al., 2004). In another study, patterned human cardiomyocytes differentiated from pluripotent stem cells (hPSC-CMs) exhibited improved contractile activity when cultured on substrates of physiological stiffness (Ribeiro et al., 2015).

#### Substrate Stiffness

##### Crosslinking during polymerisation

Biochemical crosslinking using EDAC forms isopeptide bonds between carboxyl and amino groups from different residues in direct contact. Several studies have crosslinked type I rat tail collagen with EDAC to produce substrates of different mechanical stiffness but identical ligand density (Tierney et al., 2009; Keogh et al., 2010; Haugh et al., 2011; Mullen et al., 2013, 2015; Mc Garrigle et al., 2016). One study demonstrated osteoblast differentiation on substrates of 1 kPa and osteoblast differentiation followed by early osteocyte differentiation on softer substrates of 300 Pa (Mullen et al., 2013).

Altered stiffness of polyacrylamide (PA) can be achieved by varying the percentage of acrylamide and bis-acrylamide in the polymerisation process (Wang and Pelham, 1998; Tse and Engler, 2001; Engler et al., 2006; Lee et al., 2013, 2014). MSCs cultured on stiff PA substrates (15 kPa) have been shown to exhibit higher expression of smooth muscle cell (SMC) markers (α-actin, calponin-1), whereas MSCs cultured on soft PA substrates (1 kPa) exhibit increased chondrogenic (collagen-II) and adipogenic (LPL) marker expression (Park et al., 2011). The same study sought to understand the effect of matrix stiffness on MSC differentiation in response to transforming growth factor beta (TGF-β). TGF-β increased expression of SMC marker on stiff substrates and increased chondrogenic marker expression on soft substrates but suppressed expression of adipogenic markers on soft substrates. Another study tailored both the peptide displayed to cells and the substrate mechanical properties and in this way generated PA hydrogels that bind human ESC (hESC) surface GAGs (Musah et al., 2012). They showed that hESCs can respond to mechanical information transmitted via GAG engagement, and that stiff matrices (10 kPa) activated YAP/TAZ nuclear localisation, whereas this was not observed on softer (0.7 kPa) substrates. It was proposed that stiff substrates are more effective for long-term self-renewal of hESCs. In another study, osteogenic differentiation of BMSCs on stiff substrate (15–40 kPa) was inhibited by depletion of YAP and TAZ, culturing cells on soft substrate (0.7–1 kPa) or incubating with a Rho inhibitor (C3) (Dupont et al., 2011). Interestingly, YAP and TAZ knockdown allowed adipogenic differentiation on stiff substrates by mimicking a soft environment.

Polyethylene glycols crosslinking is compatible with cell encapsulation and maintenance of cell viability, which facilitates tuning mechanical properties in the presence of living cells (Liang et al., 2011). The approach involves combining varying amounts of PEG−diacrylate (PEGDA) with non−acrylated PEG, and the substrates must be modified or coated to enable cell attachment. Dynamic substrate stiffening has been implemented to mimic *in vivo* changes in temporal stiffness, by means of thiolated-hyaluronic acid (HA) hydrogels crosslinked with PEGDA, whereby their stiffness was modulated by varying crosslinker molecular weight (Young and Engler, 2011). Pre-cardiac cell seeded collagen-coated HA hydrogels increased expression of mature cardiac markers and formed more mature muscle fibers than when grown on compliant PA hydrogels.

Embryonic stem cells were grown on collagen I coated PDMS substrates of varying stiffness (0.041–2.7 MPa) achieved by varying crosslinker concentrations (1–23% (w/w)). It was reported that osteogenic differentiation (OPN and RUNX2 expression) and mineralisation by ESCs was enhanced on stiff substrates (>2.3 MPa) when compared to soft substrates (0.04–1.9 MPa) (Evans et al., 2009), and genes expressed in early mesendoderm differentiation were also upregulated. Cell spreading and growth increased as a function of substrate stiffness, whereas cell attachment was unaffected.

Human HSCs cultured on tropoelastin substrates (stiffness not reported) enhanced expansion and maintenance of undifferentiated cells (Holst et al., 2010). When the substrates were cross-linked with glutaraldehyde at concentrations greater than 0.1% to alter the elasticity (not reported) the biological effects of tropoelastin were lost and mechanotransduction inhibition also abrogated these effects.

Other polymers including poly(propylene fumarate) (PPF) (Payne et al., 2002) and polymethylmethacrylate (PMMA) (Dalby et al., 2007) have also been used, both of which can have their stiffness controlled through crosslinking during the polymerisation process.

##### Photopolymerisation

Neural stem/progenitor cells cultured on photopolymerizable methacrylamide chitosan substrates were found to be most proliferative on soft substrates (<10 kPa). Neuronal differentiation was favored on soft surfaces (<1 kPa) whereas oligodendrocyte differentiation occurred on stiffer surfaces (>7 kPa) (Leipzig and Shoichet, 2009). Astrocyte differentiation was only observed in a small percentage on substrates less than 1 and 3.5 kPa.

##### Ligand availability

Extracellular matrix stiffness can also be altered by coating cytotoxic polymers with cell adhesive ligands such as collagen (Evans et al., 2009), laminin (Rowlands et al., 2008) and fibronectin (Rowlands et al., 2008; Altmann et al., 2011). The differentiation of MSCs cultured on collagen I substrates with different ligand coatings was examined (Rowlands et al., 2008). Osteogenic differentiation (RUNX2 expression) increased with substrate stiffness (from 0.7 kPa to 80 kPa), and was found to occur significantly only on high stiffness collagen I-coated PA gels (80 kPa), whereas substrates with collagen IV, fibronectin or laminin I stimulated osteogenic differentiation when the stiffness was of the order of 25 kPa. Myogenic differentiation occurred on all gel-protein combinations that had stiffness greater than 9 kPa, but peaked for fibronectin coated gels with a modulus of 25 kPa. Another study reported that increasing ECM stiffness of Matrigel coated PA hydrogels *in vitro* increases hPSC and colony spread area but did not alter self-renewal (Keung et al., 2012), which is in contrast to the findings with mESCs. Soft matrices (100 – 700 Pa) promoted expression of early neural ectoderm markers, and downstream increases in total neurons and dopaminergic neurons. A recent study explored the effects of varying stiffness (3 and 38 kPa), ECM type and ligand density on YAP nuclear translocation in hMSCs using ligand (fibronectin, collagen I, collagen IV and laminin) coated PA substrates (Stanton et al., 2019). On stiff hydrogels (38 kPa), low ligand density (5 μg/mL) resulted in YAP nuclear translocation for fibronectin, collagen I and collagen IV coated PA substrates whereas high ligand density (20 μg/mL) was necessary for YAP nuclear translocation on laminin coated PA substrates. Moreover, cytoplasmic YAP localization, observed for low ligand densities of collagen I or fibronectin coated stiff hydrogels, resulted in low osteogenic commitment (cytoplasmic RUNX2 and low ALP expression). In contrast, nuclear YAP localization resulted in nuclear RUNX2 localization and higher levels of ALP expression for all high ligand density ECM coated stiff hydrogels except collagen IV.

**FIGURE 3:**
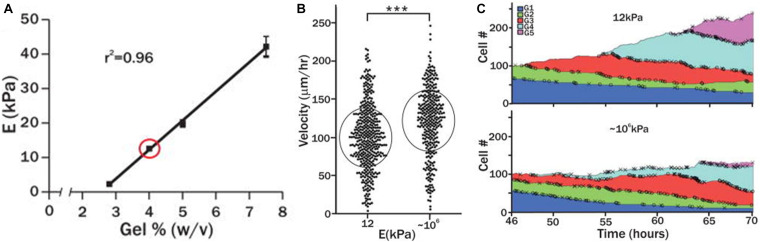
**(A)** The mechanical properties of PEG hydrogels were altered by varying precursor polymer concentration. **(B)** Velocity of skeletal muscle stem cells cultured on soft PEG hydrogel (12 kPa) or rigid cell culture plastic (106 kPa). **(C)** Number of skeletal muscle stems (normalized) cultured on soft (12 kPa) or rigid substrate (106 kPa) over the course of 70 h. Adapted with permission from Gilbert et al. (2010). ***p < 0.0001.

A tunable PEG hydrogel platform, with a range of rigidities (2–42 kPa), was developed by altering the percentage of PEG polymer (∼2.8–7.5% w/v) in precursor solution (Gilbert et al., 2010), and then laminin was used as an adhesion ligand covalently crosslinked to the hydrogel network. Skeletal muscle stem cells (SMSCs) cultured on soft PEG hydrogels (12 kPa), with stiffness close to native muscle elasticity, promoted self-renewal *in vitro* and enhanced muscle regeneration when transplanted into mice. This was not observed on stiff tissue culture plastic (∼106 kPa). In this way, substrate elasticity was shown to be a potent regulator of SMSCs fate in culture. Moreover, the migration velocity of the stem cells increased (120 μm/h) when they were cultured on the stiff PEG hydrogels when compared to softer matrices (99 μm/h) (Figure 3). One study reported that colony formation by hESCs is modulated more strongly by the wettability than by variation in the elastic moduli (Mei et al., 2010). The spatial organization of hMSCs was investigated on PEG hydrogels of varying substrate stiffness (soft (7.4–11.2 kPa) and stiff (27.3–36.8 kPa)) and ligand presentation (varying RGD concentrations (0.05–2.5 mM)) (Chahal et al., 2018). Regardless of RGD concentration and isoform, hMSCs seeded on soft PEG hydrogels clustered with reduced cell attachment and spreading area. For RGD concentrations of greater than 0.5 mM, hMSCs seeded on stiff hydrogels spread with high spatial coverage. Thus, it was proposed that both hydrogel stiffness and ligand presentation are important factors in regulating hMSC organization.

In another study hMSC seeded collagen-coated PDMS of a range of stiffness (soft, 0.07–0.10 kPa, stiff, 2.15–2.40 MPa) demonstrated diminished cell contractility on soft substrates of both hydrophobic PDMS and hydrophilic polyethylene-oxide-PDMS (PEO-PDMS). However, cell spreading and osteogenic differentiation occurred only on soft hydrophobic PDMS and not on soft hydrophilic PEO-PDMS (elastic modulus < 1 kPa) (Razafiarison et al., 2018).

##### Carbon nanotubes

Carbon nanotubes (CNTs) have high stiffness with a Young’s modulus of approximately 1 TPa (Treacy et al., 1996; Wong et al., 1997). CNTs have desirable mechanical properties for use as biomaterials in orthopedics for bone regeneration (Harrison and Atala, 2007; Tran et al., 2009) due to their good strength, elasticity and fatigue resistance, their 3D porous structure, their interlinked nano-network structure and appropriate porosity, their controllable electrical conductivity and their cylindrical shape and nanoscale dimensionality. One study demonstrated that rat BMSCs incubated on a glass surface densely coated with carbon nanotubes exhibit high ALP activity, upregulation of osteogenic markers (BMP2, RUNX2, ALPl and OCN) and increased calcium content (Mori et al., 2020). The recent application of CNT for tissue engineering through stem cell differentiation is discussed in great detail in a review paper (Lee et al., 2015).

#### Substrate Thickness

Substrate thickness has been used as a method of varying the local stiffness experienced by the cell (Sen et al., 2009; Leong et al., 2010). The mechanical influence of substrate thickness is related to cell contractility, whereby on thin substrates (<5 μm) contractile forces produced by the cells can propagate throughout the entire substrate and in these cases the underlying material govern the mechanical environment presented to the cells and thus cell behavior. On thicker substrates the forces cannot propagate throughout the substrate and thus the cell behavior is governed by the substrate material properties. This approach allows for investigation into the role of substrate stiffness without altering substrate composition. The effect of substrate stiffness was demonstrated in one such study, which investigated the differentiation of MSCs into various phenotypes when cultured on PA substrates of varied stiffness (0.1 – 40 kPa), achieved by varying the substrate thickness (Engler et al., 2006). Cells on the softest substrates (0.1 – 1 kPa) were found to mimic brain neurite cells, demonstrating a branched morphology. Those cultured on the intermediate stiffness substrates (8 – 15 kPa), similar to muscle tissue, displayed a spindle like morphology, whereas when MSCs were cultured on substrates similar to non-mineralised osteoid (15 – 40 kPa) they developed a spread morphology similar to osteoblasts. The differentiation profiles were confirmed by upregulation of neurogenic, myogenic and osteogenic specific markers, respectively. In another study that investigated the effect of substrate stiffness, by varying substrate thickness, it was shown that MSCs cultured on collagen coated PA gels of 0.5 mm exhibited the same spread morphology as those cultured on collagen substrates of 34 kPa. Those cultured on thicker substrates (2 mm) of identical composition were shown to behave similarly to MSCs cultured on 1 kPa collagen gels (Amnon et al., 2010). Substrate stiffness has also been shown to effect differentiation of MSCs seeded on collagen I gels with varying thickness (>10 μm – 500 μm). It was shown that MSCs on soft (thick) substrates had a lower expression levels of SMC markers (α-actin and calponin-1) than MSCs grown on a thin (stiff) substrate (Park et al., 2011). Chondrogenic marker (collagen II) increased in human MSCs grown on thick gels (soft).

Wedge shaped gels have also been used to vary substrate thickness (Merkel et al., 2007; Rudnicki et al., 2013), and have shown that cell area and traction were influenced by substrate stiffness. However, substrate microstructure also governs the effect of substrate thickness. Specifically, it has been demonstrated that the focal adhesion area decreases as substrate thickness increases (up to 5 μm thickness) and cell induced forces travel only a limited distance (micrometers) through linear, homogenous substrates such as PA (Maloney et al., 2008). Cells on fibrous substrates can be influenced by structures that are up to 130 μm away (Leong et al., 2010; Feng et al., 2013). The fibrous nature of biological substrates enables cell-induced forces to propagate through individual fibers to interact with the underlying coverslip (Rudnicki et al., 2013).

#### Substrate Rigidity Gradients

**FIGURE 4:**
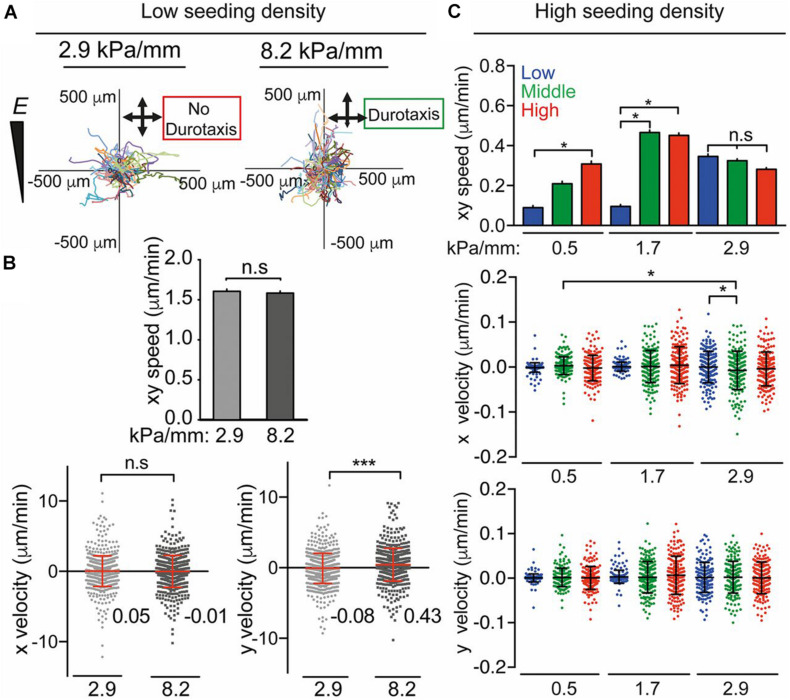
**(A)** Human ASC migration and **(B)** speed/velocity on 2.9 kPa/mm and 8.2-kPa/mm stiffness gradient fibronectin-coated PA gels over 72 h. **(C)** Average speed (xy) and x and y velocity over 72 h of hASCs on low (0.5 kPa/mm), middle (1.7 kPa/mm), and high (2.9 kPa/mm) stiffness gradient hydrogels. Adapted with permission from Hadden et al. (2017). *P < 0.05, ***P < 0.001.

Cells are found to migrate toward areas of higher stiffness, a process known as “durotaxis,” and focal adhesion traction is critical to this process (Plotnikov et al., 2012). Durotaxis is the term used to describe cell migration governed by rigidity gradients arising from the microstructural properties of the substrate, and typically involves cell migration preferentially towards stiffer substrates (Lo et al., 2000; Cukierman et al., 2001; Zamir and Geiger, 2001; Saez et al., 2005; Schwarz and Bischofs, 2005). By altering the differential diffusion distance of cross-linker and monomer into a PA hydrogel it was possible to produce substrates with stiffness gradients (0.5, 1.7, 2.9, 4.5, 6.8, and 8.2 kPa/mm) at the cell–matrix interface. Stiffness-dependent human adipose-derived stem cell (hASC) morphology, migration, and differentiation were studied (Hadden et al., 2017). Stiffness gradients of 2.9 kPa/mm were found to be nondurotactic, whereas durotaxis was observed on matrices with gradients of 8.2 kPa/mm (Figure 4). The mechanosensitive proteins Lamin A/C, Lamin B, YAP and myocardin-related transcription factor (MRTF-A) were analyzed. Lamin A expression scaled in a dose-dependent manner in response to stiffness, and Lamin A/Lamin B ratios increased exponentially with stiffness. Nuclear translocation of YAP was confirmed to be sensitive to stiffness for certain ranges. The MRTF-A was affected by stiffness and peaked at ∼20 kPa. Adipogenic marker PPARγ was upregulated at 3 kPa, myogenic transcription factor MyoD was upregulated at 12 kPa; whereas the osteogenic marker CBFA1 was highest at 36 kPa.

Human MSCs have been investigated on hydrogels of varying stiffness gradients (0.04–1.6 kPa/μm) and different absolute stiffness (2.5–10 kPa) prepared using photo-crosslinkable gelatins (Moriyama and Kidoaki, 2019). For every increase in the absolute stiffness (2.5, 5, and 10 kPa), the threshold stiffness gradient (TG) also increased (0.14, 1.0, and 1.4 kPa/μm, respectively). This was because the cells formed more stable focal adhesions in the stiffer region, as confirmed by vinculin staining. They concluded that the intrinsic stiffness gradient of the material must be greater than position-dependent stiffness to induce cellular durotaxis.

#### Substrate Stiffness and Porosity

To uncouple the role of matrix tethering, matrix porosity and matrix stiffness for governing stem cell differentiation, a study modulated substrate porosity in PA gels without altering modulus (Wen et al., 2014). It was shown that increasing the concentration of the bis-acrylamide crosslinker could achieve substrates with stiffness in the range of 4–33 kPa, and also that specific formulations of crosslinker decreases the relative pore size without markedly altering hydrogel modulus. This study reported that varying substrate porosity, by altering the ratio of acrylamide monomer and bis-acrylamide crosslinker, did not significantly change matrix tethering, substrate deformations or stem cell differentiation potential. Moreover, osteogenic and adipogenic differentiation were unaffected by varying the protein–substrate linker density or in the absence of protein tethering. Thus, it was proposed that matrix stiffness regulates stem cell differentiation independently of protein tethering and porosity (Wen et al., 2014).

Another study sought to understand the combined influence of matrix stiffness and porosity on the fate of human epidermal stem cells and MSCs by culturing them on collagen coated PDMS surfaces that had been crosslinked to achieve a range of substrate stiffness (0.1 kPa – 2.3 MPa) and PA hydrogel surfaces of varying stiffness (0.5–740 kPa) (Trappmann et al., 2012). For the PA surfaces, pore size was inversely correlated with stiffness, resulting in substantial differences in effective porosity for the varying stiffness substrates (15 nm in 2 kPa gels, but >2 nm for the stiff gels above the 115 Pa). Most interestingly, epidermal stem cells plated on the most porous substrates were rounded and underwent terminal differentiation, whereas on low porosity substrates (stiffest gels) cells spread and remained undifferentiated. It was proposed that porosity influences cell attachment because the collagen anchoring points would be further apart in softer gels. These findings support the concept that matrix stiffness does not govern cell shape in 3D hydrogels, and that stem-cell differentiation is regulated by the ability of cells to remodel the ECM. Moreover, it was shown that stem-cell fate can indeed be determined by the collagen anchoring density whereby keratinocytes differentiated and did not spread when grown on collagen with a low nanoparticle density (190 nm spacing), but cells spread and did not differentiate when cultured on collagen with closely anchored nanoparticles (60 nm). It was concluded that epidermal stem cells and MSCs exert a mechanical force on collagen coated substrates and consequently respond to the mechanical feedback provided by the collagen. This feedback is altered in hydrogels of different stiffness due to variations in anchoring densities, and ultimately governs stem cell-fate decisions (Trappmann et al., 2012).

#### Switching Stiffness – Mechanical Memory

The culture of MSCs on soft (∼0.5 kPa) or stiff (∼40 kPa) hydrogels followed by transfer to hydrogels of the opposite stiffness have been investigated (Lee et al., 2014). PA hydrogels of varying stiffness were developed and PDMS stamps (fabricated using photolithography based approaches) were used to pattern the PA surfaces with fibronectin. It was reported that MSCs cultured on soft gels expressed markers for neurogenesis whereas those cultured on stiff hydrogels expressed increased markers of osteogenesis. Transfer of MSCs to hydrogels of the opposite stiffness resulted in a switch in lineage specification. Of note, MSCs originally cultured on stiff hydrogels maintained increased markers of osteogenesis, suggesting a degree of irreversible activation. On this basis it was proposed that MSCs remain susceptible to matrix stiffness for several weeks and can redirect lineage specification in response to altered cues. ASCs have been shown to behave similarly to BMSCs by committing to becoming neurogenic, myogenic, and osteogenic on 1, 10, and 34 kPa PA substrates, respectively. Moreover, lineage-specific mRNA expression was higher in ASCs than BMSCs. Interestingly ASCs fused into multi-nucleated myotubes and expressed mature muscle proteins and remained fused even when switched to a stiff niche, which had not been previously reported for BMSCs (Choi et al., 2012).

Photodegradable PEG hydrogels (∼10 kPa) were altered to become soft (∼2 kPa) by irradiating with UV light (Yang et al., 2014). It was reported that activation of YAP/TAZ and RUNX2 in human MSCs cultured on soft substrates (2 kPa) depended on previous culture time on stiff substrates (3 GPa). Moreover, a threshold duration for mechanical priming was uncovered, whereby MSCs cultured initially for short durations (>7 days) on stiff PEG hydrogels, followed by culture on soft phototunable PEG hydrogels, demonstrated reversible activation of YAP/TAZ and RUNX2. This activation was irreversible in cells cultured for 10 days on stiff phototunable PEG hydrogels before further culture on soft substrates. It was also reported that increased durations of culture for MSCs on stiff tissue culture polystyrene enhanced osteogenic differentiation. On the basis of these findings it was proposed that stem cells possess mechanical memory of past physical environments, mediated by YAP/TAZ signaling, which influences stem cells fate. Using methacrylated HA substrates fabricated using addition polymerisation and crosslinked using Dithiothreitol (DTT), UV light was used to switch substrates from soft (3 kPa) to stiff (30 kPa) (Guvendiren and Burdick, 2012). The immediate and long-term response of human MSCs to this *in situ* substrate stiffening reported that hMSCs switched from adipogenic to osteogenic differentiation upon such stiffening. These changes were accompanied by increases in cell area, traction forces and motility, which equilibrated within 2–4 hours. Notably, the timing of the change in the mechanical environment was critical, whereby early switching (minutes-to-hours) favored osteogenic differentiation of the hMSCs whereas later switching (days-weeks) tended towards adipogenesis.

#### Micropost Arrays

Micropost arrays (MAs) can be used to obtain various mechanical rigidities for the purposes of studying mechanobiology. The arrays are composed of deformable elastomeric materials, onto which contractile cells are seeded. The cells exert traction forces to deflect the posts and, by Beam Theory, the elasticity (E), height (L) and diameter (d) of these posts determines the degree to which they bend in response to these forces. For such studies, master molds are made of materials such as silicon. MAs are developed by forming copies of this master mold, using silicon or thermoplastic material such as PDMS (Tan et al., 2003). By varying the height of the posts in the manufacturing process, this approach allows for investigation of substrate rigidity independent of surface properties.

Micromolded PDMS hexagonal microarrays (PMAs) were constructed using microfabricated silicon masters (Fu et al., 2010) and the length of PDMS microposts was varied to alter substrate rigidity (high rigidity: *L* = 0.97 μm, *K* = 1,556 nN/μm, medium rigidity: *L* = 6.1 μm, *K* = 18.16 nN/μm, low rigidity: *L* = 12.9 μm, *K* = 1.90 nN/μm), while maintaining the same surface geometry. Using these PMAs hMSC differentiation was investigated in relation to substrate stiffness. Through histological staining and PCR gene expression analyses it was reported that osteogenic differentiation was favored on rigid PMAs (*K* = 1,556 nN/μm) whereas adipogenic differentiation was enhanced on softer arrays (*K* = 1.90 nN/μm). There was a strong correlation between traction forces and the differentiation of hMSCs, whereby cells that underwent osteogenic differentiation demonstrated higher traction forces than non-differentiating cells and hMSCs that did not differentiate into adipocytes were more contractile than differentiating adipocytes. This study also reported that osteogenesis of hMSCs was decreased following treatment with Y-27632 to inhibit Rho-ROCK signaling, which provided evidence that osteogenic differentiation on stiff substrates is mediated by the actin cytoskeleton. In another study, microposts were constructed using silicon MA masters and deep reactive ion etching of the silicon masters was conducted for varying durations to achieve different micropost heights (Sun et al., 2012). PDMS was poured over the master and cured to obtain a mold from which the PDMS micropost array was fabricated and this was then coated with vitronectin to promote cell adhesion. This approach achieved a range of effective stiffness (1.92 kPa – 1,218.4 kPa). Using this PMA system it was reported that substrate rigidity plays an important role in regulating stem cell pluripotency, where greater than 20% of hESCs cultured on rigid PMAs (1218.4 kPa) remained undifferentiated compared to cells on soft PMAs (1.92 kPa). Moreover, hESCs were shown to increase cytoskeletal contractility with increased matrix rigidity. It was proposed that cytoskeleton contractility in response to changes in matrix properties might be associated with gap junctions. In another micropost array study, it was reported that neural induction of hESCs can be accelerated by altering the micropost stiffness, whereby a soft substrate (5 kPa) promoted hESC neuroepithelial conversion (Sun et al., 2014). Moreover, the purity and yield of functional motor neurons derived from these neural progenitors was enhanced on soft (5 kPa) compared to rigid (1,200 kPa) PMAs. Through immunofluorescent staining and Western Blot assays, it was shown that this process involved Smad phosphorylation and nucleocytoplasmic shuttling, regulated by Hippo-YAP signaling and cytoskeletal contractility.

### 3D Biomaterial Scaffolds

3D culture systems mimicking *in vivo* architecture and biological roles of the ECM surrounding encapsulated cells recapitulate the *in vivo* environment to a degree of complexity not achievable in a 2D culture system. Human MSCs were seeded on fibronectin-hyaluronic acid (FN–HA) hydrogels containing different amounts of FN (Trujillo et al., 2020). In the absence of FN, YAP mainly localized in the cytoplasm, whereas in the presence of FN (50 μg/mL to 500 μg/mL), YAP mainly localized in the nucleus. This was observed even though the elastic modulus was similar across all formulations (7 kPa). In contrast YAP translocation to the nucleus did not increase with increasing amounts of FN in 3D. Thus it was proposed that YAP nuclear translocation is affected by dimensionality and by cell spreading in 3D compared to 2D, as cell spreading was similar across all experimental groups. There are a vast number of papers investigating 3D biomaterial systems for stem cell culture and here we limit our discussions to crosslinked, degradable, porous and viscoelastic biomaterials in order to further understand how mechanical properties of the biomaterial matrices effect stem cells. For detailed discussions about biomaterial properties and their effects on stem cells the readers are referred to read reviews (Dawson et al., 2008; Kraehenbuehl et al., 2011; Zhao et al., 2019). Tissue engineering scaffolds provide the cells with a 3D platform for cell attachment and proliferation, whilst also providing the mechanical stability needed to deal with the physiological and biological challenges in the *in vivo* environment. To achieve access and space for the cells to proliferate and make a matrix, but also to allow for nutrient supply, most 3D biomaterial scaffolds are developed to be porous. This porosity and 3D architecture can be incorporated by various approaches, including freeze drying, electrospinning, extracting porogen templates using solvents, degradation of soft materials, or 3D printing. It should be noted that these provide biomaterials with complex 3D mechanical environments, which are dictated by the stiffness of the bulk material from which the scaffold is comprised, as well as the topographical features of the material and the specific characteristics of the porous architecture (e.g., porosity, pore size, strut thickness).

The extent to which ECM rigidity affects stem cell phenotype has been investigated in a 3D culture system where MSCs were encapsulated in PEGDM polymers at varying weight percent, which were photo-crosslinked in the presence of acryloyl-PEG-GRGDS2 (Huebsch et al., 2010). Osteogenic differentiation occurred predominantly when MSCs were encapsulated within moderate stiffness 3D hydrogels (11–30 kPa), whereas adipogenesis was favored for hydrogels within the 2.5–5 kPa stiffness range. Contrary to 2D *in vitro* culture studies, it was reported that stem cell and nuclear morphology were not strongly correlated to the mechanical properties of the 3D hydrogels for the specific ranges investigated. However, matrix stiffness regulated integrin binding and reorganization of adhesion ligands through cell contractility and blocking RGD binding to integrins inhibited osteogenesis. Adhesion, shape, and cytoskeletal organization of MSCs were shown to depend on the stiffness (0-40 kPa) of 3D cross-linked hyaluronic acid (HA) and 2D PA substrates, with stiffer matrices promoting cell spreading (Rehfeldt et al., 2012). Stem cells embedded in HA matrices were constrained to spherically symmetric shapes and the assembly of a predominantly cortical cytoskeleton. Inhibition of myosin-II contractility (using Blebbistatin) prevented spreading of MSCs treatment. Human BMSCs were encapsulated in collagen scaffolds of different stiffness (1, 2, 7 and 29.7 kPa) and cultured in the presence of 1:1 pro-osteogenic (50 μ/mL ascorbic acid, 10 mM β-glycerophosphate and 100 nM dexamethasone) and pro-adipogenic (1 μM dexamethasone, 200 μM indomethacin and 0.5 mM 3-Isobutyl-1-methyl-xanthine) media to investigate the adipogenic and osteogenic differentiation potential of hBMSCs (Herrera et al., 2019). Both osteocalcin and perilipin were found intracellularly for all stiffness using immunohistochemical methods. These images were evaluated using custom-made macros developed for ImageJ. Medium-stiffness scaffolds (2 kPa and 7 kPa) resulted in higher osteocalcin signal intensity compared to the low (1 kPa) and high (29.7 kPa) stiffness scaffolds. With increasing stiffness (1 to 29.7 kPa), perilipin signal intensity decreased.

Interestingly it has been shown that MSCs undergo adipogenic differentiation when they are encapsulated within covalently crosslinked non-degradable HA matrices of varying stiffness (4.4–91 kPa), whereas osteogenic differentiation was observed in HA matrices that were modified to be degradable (Khetan et al., 2013). It was proposed that that hydrogel structural cues provided by covalent crosslinking mediate MSC differentiation. Within hydrogels of the same modulus, osteogenesis was favored when cells were able to contract the surrounding matrix, whereas adipogenesis was favored when cells were restricted to be rounded by secondary physical crosslinking. This secondary crosslinking reduced hydrogel degradation, suppressed traction, and resulted in a change from osteogenesis to adipogenesis. In another study, MSCs were encapsulated into a nano-porous hydrogel that formed pores after injection into host tissues (via hydrolytic degradation), with the objective of decoupling pore formation from elasticity (Huebsch et al., 2015). Cell proliferation and osteogenic differentiation (ALP) were shown to peak in void-forming hydrogels with intermediate bulk stiffness (60 kPa), but drop off for those at higher stiffness (110 kPa). Collagen I expression and mineralization by MSCs within void-forming hydrogels were also shown to be enhanced in hydrogels with a bulk elasticity greater than 60 kPa. A recent study used macro-porous substrates (recombinant elastin-like protein (ELP)) that could control mechanical properties and ligand chemistry independent of each other (Haugh et al., 2018). Interestingly, MSCs upregulated markers for both osteogenesis (ALP) and adipogenesis (triglyceride) when cultured in stiff 3D porous substrates (16 and 50 kPa), when compared to soft counterparts (0.5 kPa), which diverges from previously observed responses to substrate stiffness. It was proposed that this was due to the importance of topography as a determinant of cellular behavior. One study aimed to investigate the precise effect of pore size (100, 200, and 300 μm) within 3D fibrous ECM-like scaffolds, fabricated using melt electrowriting (MEW), on the osteogenic potential of hBMSCs (Brennan et al., 2019). Human BMSCs were seeded onto MEW scaffolds and assessed to determine an optimum pore size. They found a pore size of 100 μm to be optimal demonstrating the greatest stiffness (∼0.6 N/mm), greatest seeding efficiency (55.7%) and maintenance of spread cell morphology. Interestingly, the benefits of 100 μm square pores, a pore size traditionally reported as a lower limit for osteogenesis, illustrated enhanced osteogenic effects with significantly greater collagen and calcium deposition.

The viscoelastic behavior of natural extracellular matrices was recapitulated by developing a method to alter the rate of stress relaxation of 3D hydrogels, independent of stiffness, degradation and ligand density (Chaudhuri et al., 2016). The nanoscale architecture of alginate hydrogels was modified to develop constructs with a wide range of stress relaxation rates, but a similar initial elastic modulus. Different molecular weight polymers and crosslinking densities of calcium, which ionically crosslinks alginate, were used to alter the stress relaxation properties of the hydrogels. The rate of stress relaxation was increased by (1) lowering the molecular weight of the alginate from 280 kDa to 35 kDa and (2) coupling 5 kDa PEG spacers to the 35 kDa alginate. This increased rate of stress relaxation mimics the stress relaxation rates known to be exhibited by various tissues and relevant to cell behavior. Viscoelastic alginate hydrogels that exhibited fast stress relaxation were shown to enhance cell spreading, proliferation, osteogenic differentiation by MSCs and formation of a mineralized matrix. Moreover, it was shown that these effects were mediated by integrins, local clustering of RGD ligands, actomyosin contractility, and nuclear localization of YAP. Another study investigated the role that cell volume plays in regulating stem cell fate in 3D culture. MSCs cultured in viscoelastic hydrogels demonstrated volume expansion through cell spreading which increased osteogenesis. A reduced rated of stress relaxation or an increased osmotic pressure restricts volume expansion and reduces osteogenesis, regardless of cell morphology. On the contrary, a reduced osmotic pressure induces volume expansion and accelerates osteogenesis. TRPV4 was identified as a mechanosensor of matrix viscoelasticity that regulates osteogenesis (Lee et al., 2019). It was found that TRPV4 ion channel activation and volume expansion controls nuclear localization of RUNX2 to promote osteogenic differentiation. Quantitative micro-elastography (QME) of hASCs encapsulated in 3D GelMA hydrogels demonstrated elevated cell and extracellular elasticity in 3D. Interestingly, there was an observed increase in elasticity (>10 kPa) in GelMA containing TAZ-activated hASCs (Hepburn et al., 2020).

Using microphotopatterning (μPP) substrates with aligned cell-adhesive cues, TRPV4-mediated Ca^2+^ signaling in hMSCs has been shown to be critical to the formation of aligned collagen matrix assembly (Gilchrist et al., 2019). This process can be manipulated by altering TRPV4 activity such that inhibition of TRPV4 reduced Ca^2+^ signaling and inhibited aligned collagen fibril assembly and activation of TRPV4 accelerated aligned collagen formation. TRPV4-dependent Ca^2+^ oscillations were found to be independent of pattern shape or subpattern cell location (Gilchrist et al., 2019). A FRET-based intracellular tension sensor was used to examine the effects of TRPV4 activity on tension across the protein vinculin within focal adhesions and inhibition of TRPV4 decreased tensile force across vinculin, whereas activation of TRPV4 resulted in a dynamic unloading and reloading of vinculin. Thus it was proposed that, in combination with substrate-mediated control of cell shape and position, TRPV4-dependent Ca^2+^ signaling in MSCs regulates aligned collagen fibril assembly.

### Mechanical Loading

It is widely understood that other forms of mechanical stimulation, such as fluid shear stress, hydrostatic pressure and tensile strain also influence the fate of MSCs *in vitro* (Koike et al., 2005; Sen et al., 2008; Arnsdorf et al., 2009; Kearney et al., 2010; Case et al., 2011). For example, intermittent and oscillatory fluid flow can induce osteogenic expression (calcium signaling, osteopontin and osteocalcin expression) of bone osteoprogenitors (Kreke and Goldstein, 2004; Li et al., 2004). Cyclical mechanical strain (8%) increases markers of osteogenesis (ALP, OC, Col I, Col III, Cbfa1) in human BMSCs (hBMSCs) (Jagodzinski et al., 2004). In another study, 10% cyclic mechanical strain has shown to stimulate higher amounts of ALP and calcium deposition by dental MSCs via RANKL activation. It also showed dramatic changes in mRNA and protein expression of osteogenesis-specific biomarkers, such as OPG, BSP and DSP (Zhang et al., 2019). Application of hydrostatic pressure, within ranges similar to that seen *in vivo* (0.1 MPa – 10 MPa), can enhance chondrogenic differentiation of stem cells in aggregates or seeded on collagen or agarose scaffolds, and thus promote the production of the cartilage template (Angele et al., 2003; Miyanishi et al., 2006; Finger et al., 2007; Luo and Seedhom, 2007; Wagner et al., 2008; Ogawa et al., 2009; Meyer et al., 2011; Vinardell et al., 2012; Carroll et al., 2014; Freeman et al., 2017). Furthermore, mechanical stimulation results in increased mineralized matrix production by hBMSCs cultured in 3D, short bouts of dynamic compression (5%) induce bone matrix production (Sittichokechaiwut et al., 2010). In another study, hBMSCs were encapsulated in alginate (Alg)/HA or Alg/hydroxyapatite (Hap) hydrogels (Schiavi et al., 2018). Hydrogels were cultured for 28 days and stimulated daily. Mechanical loading increased chondrogenesis in Alg/HA hydrogels, with presence of GAG and collagen II. In the Alg/Hap hydrogels increased collagen X was detected. It was proposed that Hap induces stem cells to differentiate into a hypertrophic chondrocytic phenotype and increased mechanical strength of the hydrogel. The mechanical behavior of the stratified hydrogels were investigated by plane–strain compression tests. Interestingly, it has been reported that increasing hydrogel stiffness from 5 kPa to 29 kPa restricted hMSC spreading in 3D GelMA hydrogels, whereas cyclic compressive strain (0.15 to 0.63 mm) increased cell spreading. Furthermore, the highest strain (42%) group showed a significant increase in osteogenic differentiation (RUNX2 expression and calcium deposition) of hMSCs in 5 kPa GelMA hydrogel compared to other groups (Seo et al., 2018).

Substrate stiffness can be altered by the application of extrinsic mechanical loading to the material. A four point bending device enabled the tuning of substrate stiffness by applying microstrain tensions to cell-seeded substrates (Qi et al., 2008). However, such approaches also change the shape of the substrate, which may affect cell behavior independent of substrate bulk material properties (Marcello Pilia et al., 2013). One study found that application of low intensity vibration (LIV) restored MSC proliferation and nuclear proteins LaminA/C and Sun-2 when subjected to simulated microgravity (sMG) (Touchstone et al., 2019). Disabling LINC functionality via co-depletion of Sun-1, and Sun-2 prevented restoration of cell proliferation by LIV. Another study found that application of high magnitude high frequency (HMHF, 2.5 ***G***_peak_, 100 Hz) vibration to hASCs on a tissue culture plastic in basal and osteogenic culture media resulted in decreased osteogenic media induced changes in nuclear size and elongation (Halonen et al., 2020).

A dynamic topographic substrate was developed using a polyelectrolyte multilayer (PEM) coated shape memory polymer (SMP), which upon change in incubation temperature transitions from a flat-to-wrinkle configuration inducing a change in morphology but no change in stiffness (Sun et al., 2020). The specific objective was to investigate the progressive remodeling of human iPSC-CMs seeded onto SMP-PEM substrates within a 24-hour period. Initial wrinkle formation occured (Hour 0) in response to a change in incubation temperature from 30°C to 37°C. The alignment of hiPSC-CMcells remained unchanged early in the culture period (0–12 hours), but slowly reoriented to the wrinkle direction after hour 16. With regards to intracellular myofibril reorganization, the sarcomere index decreased early in the culture period and vinculin length decreased at early time points (hour 4 and 8) but returned to the original length at hour 12. Thin filament length and the sarcomere length increased late in the culture period (16–24 h) (Sun et al., 2020). Thus it was proposed that hiPSC-CM processes respond to dynamic structural cues from cell microenvironment.

## Characterizing Cell Contractility as a Function of Biomaterial Stiffness

To understand the intracellular mechanisms by which cells interact with surrounding matrices, it is necessary to quantify the mechanical forces exerted by cells on their underlying matrix. Microposts can be used as microscopic force sensors. Cells seeded onto these dense arrays of micro pillars (diameter 1–10 mm, and length 10–100 mm) exert traction forces, causing the pillars to bend. Each pillar acts as a cantilever beam, and thus Beam theory can be applied to estimate the contractile force from the displacement. Various PMAs (0.97–12.9 μm) were fabricated to modulate and investigate substrate rigidity (Fu et al., 2010). Finite element (FE) methods were used to predict deflections of the PMAs in response to varied horizontal traction forces. Based on these deflections, the nominal spring constant, *K*, was calculated using FEM analysis and from Euler-Bernoulli beam theory. Using this approach it was shown that hMSCs morphology, focal adhesions, cytoskeletal contractility and differentiation towards adipogenic or osteogenic lineages is governed by micropost rigidity (Fu et al., 2010).

Contractile forces exerted by cells on biomaterial substrates can also be quantified using Traction Force microscopy (TFM). The TFM technique calculates traction forces generated by cells on an underlying substrate as a function of deformations of embedded beads, which are imaged using phase-contrast or fluorescent microscopy, within the substrate under relaxed and contracted conditions (Guvendiren and Burdick, 2012). Contractile forces exerted by hMSCs on soft and stiff substrates was quantified using TFM and it was shown that hMSCs immediately respond to stiffness with increased in cell area, traction forces and motility, which equilibrate within 2–4 h.

In another important study, traction forces of ASCs cultured on PA hydrogels (4 and 30 kPa) were calculated based on displacement maps of embedded fluorescent particles resulting using traction force microscopy (Wen et al., 2014). It was shown that hydrogel deformations due to cell contractions were dependent on substrate stiffness, but not porosity. In that study AFM tip retraction velocity was matched to the pulling velocity and size of focal adhesions (Wen et al., 2014), and it was shown that cell-generated substrate displacements and cell differentiation were similar for PA–PEG–RGD hydrogels and collagen-coated hydrogels. It was proposed that this provided evidence in support of matrix-induced differentiation occurring through myosin-based cell contraction.

One study demonstrated that decreased myosin contractility governs hPSC survival and proliferation on microcarriers. (Chen et al., 2014). Human PSCs proliferated on non-coated positively charged cellulose microcarriers containing either the ROCK inhibitor (Y27632) or the myosin inhibitor Blebbistatin. Myosin phosphatase 1 and myosin light chain 2 are dephosphorylated in the presence of these two inhibitors, suggesting decreased myosin contractility.

In another study, on-chip high-throughput experiments allowed rapid assessment of the suitability of 15 methacrylated gellan-gum (GG-MA)/media combinations for the osteogenic differentiation of hASCs. Regardless of basal or growth media conditions, all hydrogel formulations resulted in the osteodifferentiation of hASCs. Moreover, the inhibition of the actin-myosin contractility pathway impaired hASCs’ osteogenic differentiation and thus it was suggested that hASC differentiation depended on the actin-myosin contractility pathway (Oliveira et al., 2016).

## Computational Modeling of Cell-Biomaterial Interactions

Computational modeling techniques can be applied to investigate the interactions between stem cells and biomaterials, which are challenging to characterize using experimental or analytical approaches due to the complex material properties and structure of the stem cells themselves, but also the biomaterial.

Finite element modeling techniques to represent and study cell mechanics were first developed by implementing the simplifying assumption that the cell could be approximated as a passive material, either by the assumption that the cell was linear elastic (McCreadie and Hollister, 1997; Charras and Horton, 2002), hyperelastic (Caille et al., 2002), viscoelastic (Karcher et al., 2003; Trickey et al., 2006) or biphasic (Guilak and Mow, 2000). Such models were applied to investigate the effect of various extrinsic mechanical stimuli, such as fluid flow (McGarry et al., 2005; Vaughan et al., 2013), externally applied strain (Stops et al., 2008; Stern et al., 2012) or strain applied directly to individual cells (Rudd et al., 2001; Mijailovich et al., 2002), on the intracellular loading state. However, biological cells are not passive but rather contract their substrate through the action of the cytoskeleton (Peterson et al., 2004; Wang et al., 2007; Guolla et al., 2012), and the resistance of a biomaterial to such contraction generates isometric tension within the cell (Goeckeler and Wysolmerski, 1995; Bodmer et al., 1997). To account for such tension, an FE model of a hyperelastic cell incorporated a compressive pre-stress throughout the cell cytoplasm for the purposes of studying the displacement and strain fields induced within the cell monolayer (Sen et al., 2009). Matrix elasticity and thickness were varied to compare deformations within the matrix. It was reported that stem cells were more sensitive to matrix stiffness than myoblasts and osteoblasts, and that cells sense their surroundings at the scale of adhesions rather than on the cellular scale (Sen et al., 2009).

Thermal contraction can simulate cell contraction and predict intracellular tension. In an unrestricted volume element, the coefficient is in effect a strain, without the associated normal or shear stresses. Under an applied boundary condition tension is generated (in the opposite direction to the strain induced by the thermal load), which is manifested by the maximum principal stress induced in the system.

**FIGURE 5:**
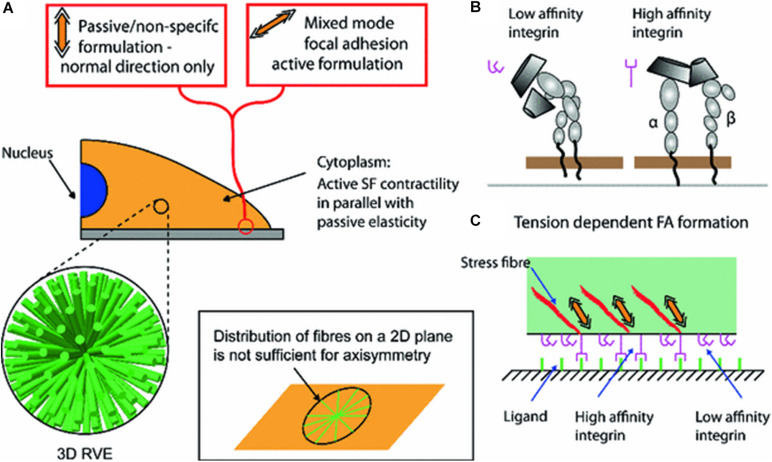
**(A)** Computational model of 3D cell with 240 fiber orientations in 3D space within a representative volume element (RVE). **(B,C)** High affinity and low affinity integrins involved in the formation of focal adhesions between a cell containing stress fibers and a ligand-coated substrate (Ronan et al., 2014).

Cytoskeletal contractility and remodeling are critical intracellular processes that enable cell-matrix interaction and stress-generation by the actin cytoskeleton throughout the cell and its matrix. An active model of cell contractility and remodeling was developed to incorporate stress fiber formation, dissociation, and contractility (Deshpande et al., 2007). This model has been used to predict the contractile responses of smooth muscle cells on a bed of microposts, and was shown to predict force exerted by cells with the number of posts and actin distributions within the cell (McGarry et al., 2009). This framework was applied to investigate stress fiber and focal adhesion formation on elastic substrates of varying stiffness (Ronan et al., 2014). It was predicted that stress fiber contractility plays an important role in the substrate-dependent response of cells, whereby compliant substrates result in dissociation of stress fibers and lower focal adhesion formation and stiffer substrates result in the presence of stress fibers and FAs (Figure 5). On compliant substrates (<2 kPa) the cell height was 6.7 μm, whereas on stiffer substrates (>100 kPa) cell height was reduced (5.3 μm). The average stress in the nucleus increased from 70 Pa on compliant substrates to 600 Pa on stiffer substrates. However, beyond a specific range of substrate stiffness (1-100 kPa) substrate stiffness did not significantly alter the stress. Cellular contractility (representative of different cell phenotypes) was predicted to alter this stiffness range. The active contractility model has also been used to examine the effects of extracellular mechanics on stress fiber formation (Ronan et al., 2012; Weafer et al., 2013) as well as the force generated by individual focal adhesions in MSCs (Ronan et al., 2013) and to simulate cell remodeling under static and dynamic loading of single cells (Dowling et al., 2012; Reynolds et al., 2014; Reynolds and McGarry, 2015).

Computational approaches have been used to investigate the precise effect of fibers on fibrous gel mechanical properties. To study the effects of fibrosity on the properties of bulk collagen gel, a microscale discrete fiber representative element was linked to a Galerkin macroscale model (Stylianopoulos and Barocas, 2007), demonstrating the non-uniform deformation of a fibrous gel. This method was further developed to investigate the response of cells to gel fibrosity (Rudnicki et al., 2013). The formation of crosslinks is dependent on the position and density of fibers in the collagen. In order for the effect of crosslinking on cell behavior to be analyzed, particular attention must be paid to the heterogeneity present in the substrate material. FE simulations were used to investigate the effects of crosslinking density and substrate thickness on the resistance of the gel to cellular forces, corresponding to the equivalent stiffness of collagen gels (Mullen et al., 2015). The models predicted that cells cultured on a soft fibrous substrate spread similar to those cultured on a stiff non-fibrous substrate. It was predicted that as crosslinking density is increased and substrate thickness decreased, an increase in equivalent stiffness of fibrous collagen gels occurs (Mullen et al., 2015).

Using, a 3D numerical model of individual cell migration, it was predicted that substrate stiffness, boundary conditions and external forces regulate cell migration (Borau et al., 2011). The model incorporated the mechanosensing process of the cell as the primary mechanism regulating its movement.

Computational modeling was implemented to probe the impact of pore topography on the mechanical stimulus that stem cells experience within 3D matrices (Haugh et al., 2018). The computational framework accounted for cellular contractility, by applying micron-displacements to cell-attachments to the matrix. The model investigated cell orientations and topography within porous substrates, which were varied to predict how these material parameters influence the local mechanical stimulus sensed by cells. It was predicted that within porous substrates cells experience heterogeneous mechanical stimuli due to the wide range of possible cellular orientations within the pores. Specifically, cells experience equivalent moduli ranging from 0.92 to 1.8 times the material modulus and that mechanical stimuli are associated with cellular orientation (Haugh et al., 2018).

Another computational model was established to investigate if substrate stiffness and oxygen tension regulate differentiation of stem cells during fracture healing (Burke and Kelly, 2012). It was hypothesized that mechanical signals act indirectly to regulate angiogenesis, that chondrogenesis of MSCs occurs in low oxygen regions, that high oxygen regions facilitate adipogenesis and that a stiff substrate facilitates osteogenesis. The model predicted all the major events of fracture repair, including cartilaginous bridging, endosteal and periosteal bony bridging and bone remodeling.

Overall, computational models have shown that different environmental stimuli are potentially integrated by stem cells *in vivo* to induce differentiation. The permissive *in vivo* environment, comprising of several growth factors and cytokines, induces differentiation of stem cells. Computational studies support the hypothesis that substrate stiffness plays an important role in determining stem cell fate in such a permissive environment. The influence of these factors alone, or in combination with extrinsic biophysical and biomechanical stimuli in regulating differentiation of stem cells requires further investigation. Understanding the key role these stimuli play is challenging using computational models alone, however by integrating computational models with appropriately designed *in vitro* and *ex vivo* studies of stem cell differentiation, their role may be elucidated.

## Discussion – Perspective and Challenges

Tissue engineering and regenerative medicine is a promising scientific field that has significant potential for treating diseases by manipulating the proliferation and differentiation of stem cells using various biochemical, biophysical and biomechanical approaches. These have provided an understanding of the biochemical environment and the desired properties of biomaterial scaffolds that are required to encourage tissue regeneration. However, the precise mechanical stimuli experienced by stem cells within these environments, and the regulatory role of such stimuli with respect to the differentiation and regeneration potential of stem cells, are still obscure.

A particular challenge is that much of the current understanding of stem cell mechanobiology has been derived from experiments on 2D monolayer cultures. While these experiments have provided an understanding of stem cell mechanotransduction, ultimately they cannot provide a complete picture of the *in vivo* situation due to limitations in the reproduction of the complex 3D cellular mechanical environment, which is dictated by the substrate stiffness, as well as the topographical features of the substrate/tissue and in many cases the addition of extrinsic loading (e.g. fluid shear stress for nutrient supply or physiological mechanical stimulation). Further fundamental studies are required to characterize the mechanical stimuli stem cells experience in their native environment, particularly the stimuli arising during regenerative processes, as this will likely govern behavior such as dormancy, potency and lineage commitment. Such studies will provide an advanced understanding of the specific biophysical cues required to regulate stem cell behavior *in vitro*. Existing stem cell-based tissue engineering approaches do not strive to mimic the *in vivo* mechanical environment surrounding these cells *in vivo* during cell renewal and specialization, primarily because these stimuli are unknown. The development of effective regenerative medicine approaches requires significant progress in understanding the precise role of mechanical stimulation in regulating stem cell renewal and differentiation *in vivo*. Such fundamental research can inform *in vitro* approaches to enhance cell-matrix interactions and tissue regeneration and ultimately enhance the development of tissue constructs for clinical applications.

The role of matrix mechanics in 3D environments has been investigated in a limited number of studies, and it has been shown that MSCs encapsulated within 3D matrices respond differently to changes in the material stiffness that those cultured on 2D substrates (Huebsch et al., 2010, 2015; Parekh et al., 2011; Khetan et al., 2013). Specifically, encapsulated cells are restricted from spreading, as cells would across a 2D substrate to enable the generation of intracellular tension. Thus in 3D environments encapsulated cells generate tension by remodeling their matrix by active degradation or by means of cellular reorganization of ligands to generate traction, and it has thus been shown that cells are thereby sensitive to changes in 3D matrix stiffness (Huebsch et al., 2010; Khetan et al., 2013; Chaudhuri et al., 2016). Cell spreading in 3D is associated with a reduction in the alignment of fibrous architectures, and is correlated with enhanced osteogenesis (Eichholz and Hoey, 2018). It has been shown that topographical features of biomaterial substrates also dictate the local mechanical environment and govern differentiation of stem cells. Specifically, highly ordered nanotopographies are not conducive to cell adhesion or osteoblastic differentiation by MSCs, whereas random and nanodisplaced nanotopographies induce osteogenic differentiation (Dalby et al., 2007). It was thus proposed that disorder may be an effective strategy for MSC differentiation. Moreover, substrates with highly aligned PCL nanofibers enhanced neuronal differentiation of ESCs (Xie et al., 2009). However, many details of the biological process whereby stem cells are governed by the 3D mechanical environment are still unclear, and further 3D *in vitro* studies are required to provide an advanced understanding of how biomaterials based approaches can be applied to govern stem cell differentiation and tissue regeneration.

Future 3D *in vitro* studies are necessary to concurrently study the interplay between mechanical cues provided by biomaterial matrices and the mechanical stimuli arising from extrinsic loading (fluid shear, compression, vibration), which will be experimentally and computationally challenging. Nonetheless, future development of effective regenerative medicine approaches requires a paradigm shift to account for the intrinsic role of all forms of mechanical stimulation for regulating stem cell renewal and differentiation *in vivo*.

While the role of surface chemistry and biochemical factors for regulating stem cell biology and tissue regeneration have been widely studied, the influence of the biophysical and biomechanical environment is less widely understood. The field of mechanobiology has developed techniques to modify and quantify mechanical properties of biomaterials, and also quantify how cells interact with such matrices. Using these techniques the role of the mechanical environment presented by biomaterial substrates and 3D scaffolds for regulating stem cell differentiation, renewal and migration have been uncovered. Computational models provide a mechanistic understanding of how biomaterial stiffness governs intracellular stimulation of the actin cytoskeleton. Future development of effective regenerative medicine approaches requires a paradigm shift to characterize and account for the crucial role of mechanical stimulation in regulating stem cell renewal and differentiation *in vivo*.

## Author Contributions

LM supervised the project. Both authors drafted the manuscript and read and approved the final manuscript.

## Conflict of Interest

The authors declare that the research was conducted in the absence of any commercial or financial relationships that could be construed as a potential conflict of interest.
